# Contribution of Membrane Vesicle to Reprogramming of Bacterial Membrane Fluidity in Pseudomonas aeruginosa

**DOI:** 10.1128/msphere.00187-22

**Published:** 2022-05-23

**Authors:** Negar Mozaheb, Patrick Van Der Smissen, Tomas Opsomer, Eric Mignolet, Romano Terrasi, Adrien Paquot, Yvan Larondelle, Wim Dehaen, Giulio G. Muccioli, Marie-Paule Mingeot-Leclercq

**Affiliations:** a Université Catholique de Louvain, Louvain Drug Research Institute, Cellular & Molecular Pharmacology Unit (FACM), Brussels, Belgium; b Université Catholique de Louvain, de Duve Institute, CELL Unit and PICT Platform, Brussels, Belgium; c KU Leuven, Department of Chemistry, Molecular Design and Synthesis, Leuven, Belgium; d Université Catholique de Louvain, Louvain Institute of Biomolecular Science and Technology, Louvain-la-Neuve, Belgium; e Université Catholique de Louvain, Louvain Drug Research Institute, Bioanalysis and Pharmacology of Bioactive Lipids Research Group, Brussels, Belgium; University of Iowa

**Keywords:** *P. aeruginosa*, bacterial membrane, membrane vesicles, fluidity, biofilm, biofilms, *Pseudomonas aeruginosa*

## Abstract

Pseudomonas aeruginosa is an opportunistic pathogen capable of resisting environmental insults by applying various strategies, including regulating membrane fluidity and producing membrane vesicles (MVs). This study examined the difference in membrane fluidity between planktonic and biofilm modes of growth in P. aeruginosa and whether the ability to alter membrane rigidity in P. aeruginosa could be transferred via MVs. To this end, planktonic and biofilm P. aeruginosa were compared with respect to the lipid composition of their membranes and their MVs and the expression of genes contributing to alteration of membrane fluidity. Additionally, viscosity maps of the bacterial membrane in planktonic and biofilm lifestyles and under the effect of incubation with bacterial MVs were obtained. Further, the growth rate and biofilm formation capability of P. aeruginosa in the presence of MVs were compared. Results showed that the membrane of the biofilm bacteria is significantly less fluid than the membrane of the planktonic bacteria and is enriched with saturated fatty acids. Moreover, the enzymes involved in altering the structure of existing lipids and favoring membrane rigidification are overexpressed in the biofilm bacteria. MVs of biofilm P. aeruginosa elicit membrane rigidification and delay the bacterial growth in the planktonic lifestyle; conversely, they enhance biofilm development in P. aeruginosa. Overall, the study describes the interplay between the planktonic and biofilm bacteria by shedding light on the role of MVs in altering membrane fluidity.

**IMPORTANCE** Membrane rigidification is a survival strategy in Pseudomonas aeruginosa exposed to stress. Despite various studies dedicated to the mechanism behind this phenomenon, not much attention has been paid to the contribution of the bacterial membrane vesicles (MVs) in this regard. This study revealed that P. aeruginosa rigidifies its membrane in the biofilm mode of growth. Additionally, the capability of decreasing membrane fluidity is transferable to the bacterial population via the bacterial MVs, resulting in reprogramming of bacterial membrane fluidity. Given the importance of membrane rigidification for decreasing the pathogen’s susceptibility to antimicrobials, elucidation of the conditions leading to such biophysicochemical modulation of the P. aeruginosa membrane should be considered for the purpose of developing therapeutic approaches against this resistant pathogen.

## INTRODUCTION

Pseudomonas aeruginosa is an opportunistic pathogen that exploits numerous strategies to maintain its homeostatic condition against environmental stress through tightly regulated cellular pathways. Biofilm formation is a strategy that enables P. aeruginosa to resist adverse environmental conditions such as exposure to antibiotics and host immune system components during infection (1, 2). During switching from planktonic to biofilm lifestyles, P. aeruginosa undergoes numerous physiological alterations, which help the bacterium to present a recalcitrant phenotype against stressors inside the biofilm. Downregulation of cellular bioenergetic processes (3), modulation of the bacterial membrane to decrease its interaction with antibiotics (4), and decrease of membrane permeability to antibiotics (5) are among the changes that the bacteria undergo to tolerate stressors. Moreover, evidence suggests that membrane fluidity is lower in biofilm bacteria (2, 6).

Bacterial changes in lipid composition and biophysicochemical characteristics of their membrane in response to stress, i.e., so-called membrane remodeling, is a prominent cell process (7). There are various mechanisms for membrane remodeling, well reviewed by Bohuszewicz et al. (7). The inner leaflet of the outer membrane, as well as the inner membrane of Gram-negative bacteria, is mainly composed of phospholipids, namely, phosphatidylcholine (PC), phosphatidylethanolamine (PE), phosphatidylglycerol (PG), and cardiolipin (CL), each consisting of fatty acyl chains (fatty acids) and a polar head (phosphatidylglycerol derivative) (1, 6). In P. aeruginosa, where straight-chain fatty acids are the predominant fatty acids of the membrane, the bacteria undergo a change in the viscosity of their membrane by altering the ratio of saturated over unsaturated fatty acids via utilizing (i) two fatty acyl desaturases under aerobic conditions, namely, DesA and DesB. The activity of these enzymes reduces the membrane rigidity. Indeed, the former introduces a *cis* double bond at the 9-position of acyl chains of phospholipids, while the latter introduces a double bond to acyl-CoA originating from exogenous fatty acids (8, 9). (ii) Altering the length of fatty acid chains (10, 11) via incorporation of longer (12) and more saturated (5) fatty acids in the structure of its membrane decreases its fluidity. The incorporation of medium- and long-chain fatty acids into the membrane of the bacteria is initiated by the conversion of fatty acids to fatty acyl-coenzyme A (CoA) via the activity of long-chain fatty acyl-CoA synthetases (FadDs) such as FadD1 and FadD2 (12). In addition to changing the structure of the preexisting lipids, *de novo* synthesis of all *cis* fatty acids is another mechanism by which alteration of membrane fluidity is achieved (13). Besides the effect of fatty acyl chains, the modulation of the PE-over-PG proportion and alteration of the abundance of CL have been described as strategies for regulating membrane fluidity in Gram-negative bacteria (1, 14).

**FIG 1:**
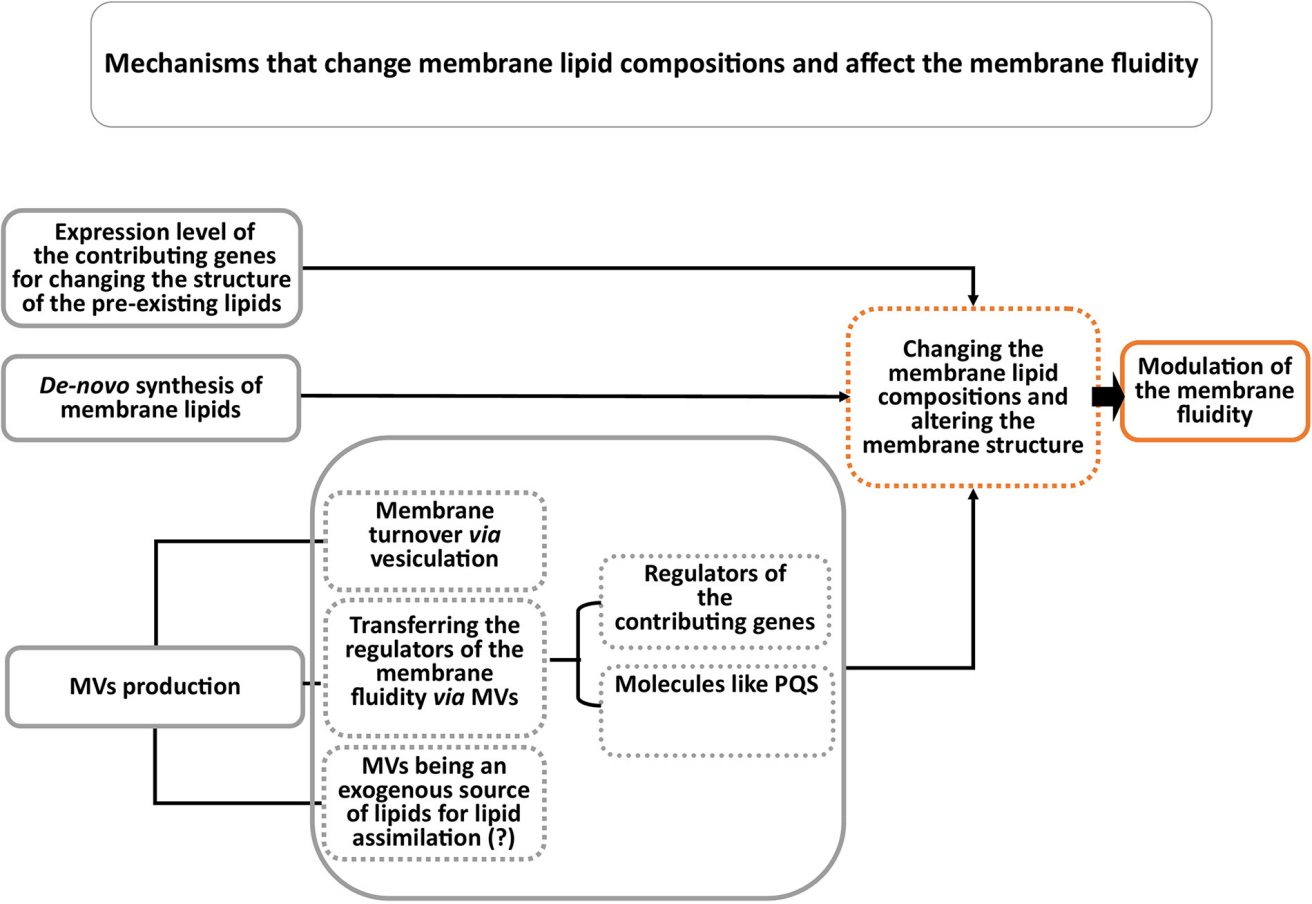
Suggested mechanisms involved in regulating membrane fluidity in P. aeruginosa.

Bacterial membrane vesicle production is another strategy of P. aeruginosa upon encountering stress conditions (15, 16). A pioneering observation by Schooling and Beveridge showed that MVs are an essential component of the biofilm matrix (17). Indeed, stresses such as nutrient depletion (18), low oxygen pressure (19), and low pH (20) trigger MV formation. Interestingly, membrane vesiculation accelerates the adaptability of bacteria to the environment by contributing to membrane remodeling (21). Concerning the regulation of membrane fluidity, MVs affect membrane fluidity by removing unwanted lipid species (14). In studies related to the function of MVs, especially in bacterial communities such as biofilms, MVs are considered to serve as public goods (22). Several pieces of evidence support this. First, in P. aeruginosa, MVs directly contribute to changing bacterial membrane fluidity by carrying regulators of membrane fluidity, such as the Pseudomonas quinolone signal (PQS) (23). Second, proteomic studies of MVs have revealed that proteins with functions in the metabolism of fatty acids and phospholipids are transported by the MVs (24, 25). Thus, the exchange of these molecules in the bacterial community could reprogram bacterial lipid metabolism. Third, some Gram-negative bacteria, such as P. aeruginosa, incorporate exogenous fatty acids into phospholipid synthesis (26). Given that MVs carry long-chain fatty acids (14, 27), they can be an exogenous source of fatty acids for the production of phospholipids with long-chain fatty acids (Fig. 1).

Appropriate membrane fluidity allows cells to respire (28), move (29), have selective membrane permeability (30), and undertake appropriate localization and functionality of membrane proteins (31). Hence, various cellular processes concomitantly contribute to maintaining membrane homeostatic fluidity. Additionally, membrane rigidification is a resistance strategy of pathogens against antimicrobial activities, although the outcome of applying this strategy is highly dependent on the antimicrobial’s mechanism of action. In Gram-negative bacteria, the outer membrane is an impermeable barrier against various molecules; however, numerous antimicrobial molecules could diffuse across the outer membrane via passive diffusion and through nonspecific porins (32). The passive diffusion of antimicrobials across the inner membrane is highly dependent on the dynamics of the phospholipids and rigidity of the membrane. A rigidified membrane provides higher protection for bacteria against the passage of antimicrobial molecules (33). Additionally, rigidified membranes possess reduced bioenergetic activity. Thus, bacteria with rigid membranes are less likely to be affected by antimicrobials (28, 34).

This study aimed to emphasize the role of MVs in the mechanisms involved in the fluidity of the bacterial membrane of P. aeruginosa PAO1 and the potential effects of MVs on biofilm formation and structure. To this end, the planktonic and biofilm P. aeruginosa and their corresponding MVs were compared in terms of their phospholipid composition. Further, the expression of genes that influence membrane fluidity were compared between the planktonic and biofilm P. aeruginosa. The fluidity maps for the bacterial membranes when the media were enriched with the MVs or not were obtained using the fluorescence lifetime imaging (FLIM) technique and a viscosity-sensitive probe (BODIPY-C10). Finally, the growth rate and biofilm formation capability of the bacteria in the presence of MVs were investigated.

## RESULTS

### MV morphology, quantity, associated mRNAs, and phospholipid composition of biofilm P. aeruginosa are different from those of planktonic bacteria.

The comparison of phospholipid compositions of P. aeruginosa’s membrane in the planktonic versus the biofilm lifestyle revealed that PE makes up a higher proportion of the membrane phospholipids. Moreover, the membrane of the planktonic bacteria has a significantly higher relative amount of PE having unsaturated fatty acids (see Fig. S1A in the supplemental material). In contrast, PE species with saturated fatty acids were relatively more abundant in the membrane of the biofilm bacteria (Fig. S1B). The differences in the planktonic and the biofilm P. aeruginosa concerning their membrane phospholipid composition (Fig. S1A, B, and C) motivated us to compare the membrane-derived vesicles (MVs) produced in these two lifestyles of the bacteria.

10.1128/msphere.00187-22.1 FIG S1 Phospholipids and gene expression analysis of planktonic versus biofilm P. aeruginosa. The proportion of phospholipids of membrane from biofilm P. aeruginosa over that of the membrane from planktonic bacteria in terms of their unsaturated fatty acyl chains (A), saturated acyl chains (B), and polar heads (C). The red dashed line shows one representing the baseline (membrane of the planktonic bacteria), and the differences in the biofilm bacteria are calculated relative to the baseline; the experiment was performed on two biological replicates. (D) Differential gene expression in the biofilm bacteria relative to the planktonic bacteria. The expression of genes was normalized against the mRNA levels of 16S rRNA, and the experiment was performed in triplicate. Data are presented as means ± SD. Multiple *t* test was used to compare the planktonic and the biofilm bacteria. ***, *P* < 0.001; **, *P* < 0.01; *, *P* < 0.05. Download FIG S1, TIF file, 1.1 MB. Copyright © 2022 Mozaheb et al. 2022 Mozaheb et al. https://creativecommons.org/licenses/by/4.0/ This content is distributed under the terms of the Creative Commons Attribution 4.0 International license.

**FIG 2:**
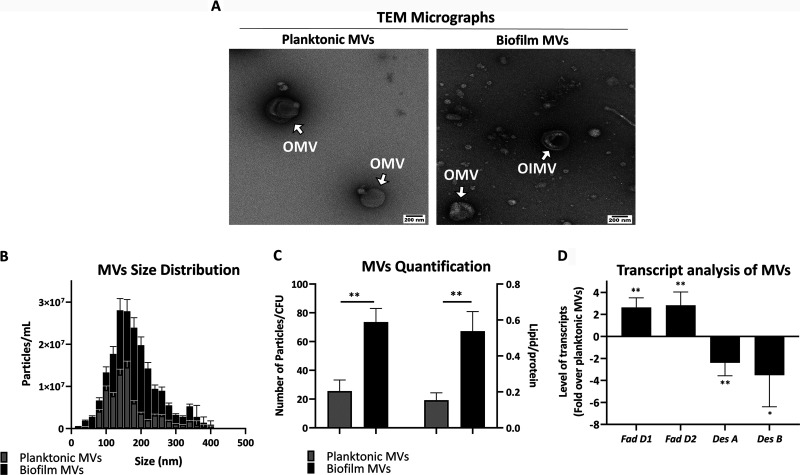
MV specifications. (A) TEM micrographs of MVs from planktonic (left) and biofilm (right) P. aeruginosa; scale bar represents 200 nm. (B) Size distributions of the MVs from planktonic and biofilm bacteria. (C) Comparative quantification of their numbers per CFU (left) and the lipid/protein proportion (right). (D) Comparison of the transcript cargos (*fadD1*, *fadD2*, *desA*, and *desB*) of MVs from the planktonic and the biofilm bacteria. The relative mRNA copy numbers were normalized against the detected templates in the samples without reverse transcriptase, and the presented data are the difference in MVs of the biofilm versus the planktonic P. aeruginosa. Data presented as means ± standard deviations (SD). Statistical analysis was performed on three biological replicates via multiple *t* test. **, *P* < 0.01; *, *P* < 0.05.

The general characterization of MVs with respect to transmission electron microscopy (TEM) micrographs (Fig. 2A), their size distribution (Fig. 2B), and quantification analysis (Fig. 2C) showed that the bacterial mode of growth (i.e., planktonic and biofilm) affects the characteristics of the MVs. In TEM micrographs, it was notable that planktonic P. aeruginosa mainly produces outer membrane vesicles (OMVs), while a large number of MVs of the biofilm bacteria were shown to have both outer and inner membrane vesicles (OIMVs) (Fig. 2A). Although the average size and the size polydispersity of the membrane vesicle population analysis showed no difference between the two groups of the MVs (Fig. 2B), the amount of vesiculation (MV particles/bacterial CFU) was almost 3-fold higher in the biofilm bacteria than in the planktonic bacteria. Moreover, the relative lipid/protein proportion of MVs of the biofilm bacteria was approximately three times higher than that of the planktonic bacteria (Fig. 2C).

Interestingly enough, targeted mRNA composition analysis of the MVs showed that MVs of planktonic and biofilm P. aeruginosa are associated with the transcripts of *fadD1*, *fadD2*, *desA*, and *desB*. It was revealed that compared with the MVs of planktonic P. aeruginosa, the MVs of biofilm bacteria were >2-fold more enriched with the transcripts of *fadD1* and *fadD2*; however, MVs of the biofilm bacteria were depleted of the mRNAs of *desA* and *desB* (Fig. 2D).

It is worth mentioning that the result of quantitative PCR (qPCR) analysis on the expression of the genes in the bacterial cells showed *fadD1* and *fadD2* are significantly overexpressed in the biofilm P. aeruginosa compared to the planktonic bacteria. Conversely, *desA* and *desB* are expressed at lower levels in the biofilm bacteria than in the planktonic bacteria. Thus, the differences between transcripts associated with the MVs could result from their differential abundance at the cellular levels (Fig. S1D). Representative amplicons of qPCR on an electrophoresis gel are provided in Fig. S2.

10.1128/msphere.00187-22.2 FIG S2 Comparative analysis of transcript levels associated with the MVs. (A) The relative transcript copy numbers of the genes of interest were normalized against the detected templates in the samples without reverse transcriptase. (B) The amplicons were loaded on agarose gel electrophoresis (2% agarose gel), and 50-bp GeneRuler was used. RT-, samples without reverse transcriptase. Download FIG S2, TIF file, 0.7 MB. Copyright © 2022 Mozaheb et al. 2022 Mozaheb et al. https://creativecommons.org/licenses/by/4.0/ This content is distributed under the terms of the Creative Commons Attribution 4.0 International license.

Additionally, we found that MVs produced by the planktonic or the biofilm bacteria possess ATP molecules, indicating that the MVs have cytoplasmic components. The amount of ATP detected in the MVs of the biofilm bacteria is roughly twice that in the MVs of the planktonic bacteria (Table 1).

**TABLE 1:** ATP quantification of MVs of planktonic and biofilm P. aeruginosa

MV sample source	ATP concn^*a*^ (nmol/10^8^ particles)
Planktonic bacteria	0.045 ± 0.005
Biofilm bacteria	0.106^*b*^ ± 0.012

a Data are averages ± standard deviations. Statistical analysis was performed on three independent experiments using a *t* test.

b *P* < 0.05.

### MVs of the biofilm P. aeruginosa are significantly enriched with long-chain fatty acids.

**FIG 3:**
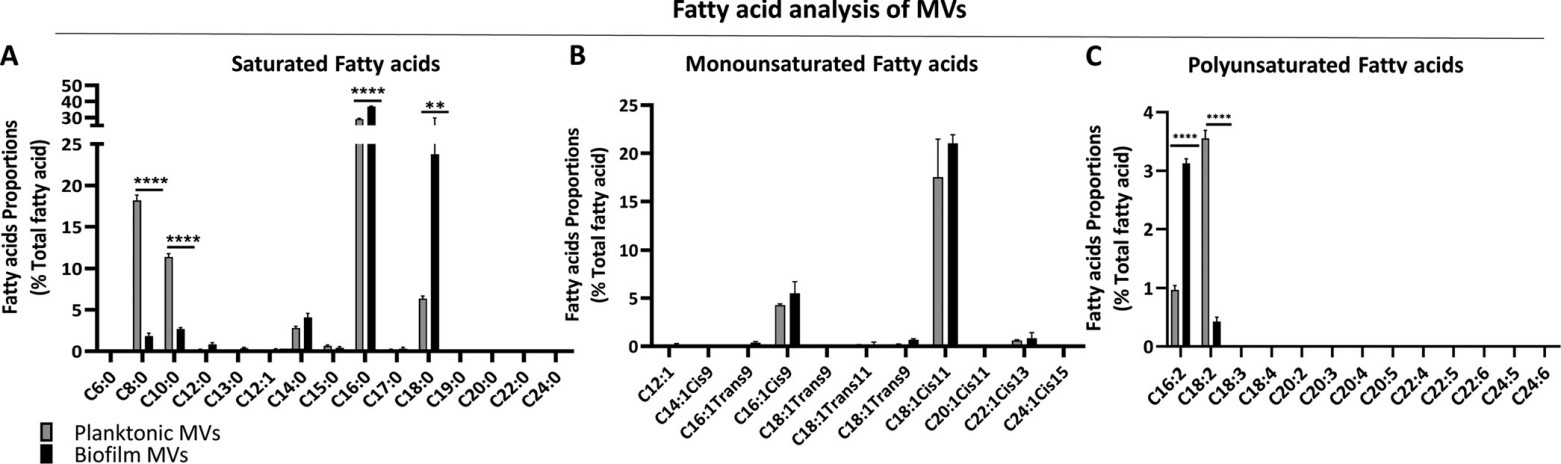
Comparative fatty acid composition of the MVs. The fatty acids are categorized into three groups, saturated fatty acids (A), monounsaturated fatty acids (B), and polyunsaturated fatty acids (C). The data are presented as means ± SD from independent replicates under each condition. Statistical analysis was performed on biological replicates via two-way ANOVA. ****, *P* < 0.0001; **, *P* < 0.01.

The fatty acid composition analysis of the MVs showed that in MVs of both the planktonic and the biofilm P. aeruginosa, more than 50% of fatty acids are saturated ones (Fig. 3A). Nonetheless, the proportions of fatty acids with the longer acyl chains (C_16_ and C_18_) are significantly higher in the MVs of biofilm P. aeruginosa than those of MVs produced by planktonic bacteria (Fig. 3A). Conversely, the MVs of the planktonic bacteria have significantly greater proportions of saturated fatty acids with shorter acyl chains, i.e., C_8_ and C_10_ (Fig. 3A). There was no significant difference between the MVs produced by planktonic P. aeruginosa versus the MVs of the biofilm bacteria regarding the proportion of monounsaturated fatty acids (Fig. 3B). Although a tiny proportion of MVs fatty acids are polyunsaturated, C_16:2_ and C_18:2_ are significantly more abundant in the MVs produced by biofilm P. aeruginosa and the planktonic bacteria, respectively (Fig. 3C).

Phospholipid composition analysis of the MVs demonstrated that MVs produced by the biofilm P. aeruginosa have a significantly higher proportion of PG than the MVs of the planktonic bacteria (Fig. S3). However, there were no remarkable differences between the MVs of planktonic P. aeruginosa versus those of the biofilm bacteria regarding the relative presence of PE and CL. Thus, differences in the phospholipid composition of the MVs are not fully in line with the originating membranes (Fig. S3).

10.1128/msphere.00187-22.3 FIG S3 Phospholipid analysis of the MVs. The proportion of phospholipids of MVs from biofilm P. aeruginosa over that of the MVs from planktonic bacteria, in terms of unsaturated fatty acyl chains (A), saturated fatty acyl chains (B), and their polar heads (C). The red dashed line shows one representing the baseline (MVs of the planktonic bacteria), and the differences in the biofilm bacteria are calculated relative to the baseline; the experiment was performed on two biological replicates. Data are presented as means ± SD. Multiple *t* test was used to compare MVs of planktonic and biofilm bacteria. ***, *P* < 0.001; **, *P* < 0.01; *, *P* < 0.05. Download FIG S3, TIF file, 1.0 MB. Copyright © 2022 Mozaheb et al. 2022 Mozaheb et al. https://creativecommons.org/licenses/by/4.0/ This content is distributed under the terms of the Creative Commons Attribution 4.0 International license.

### The MVs from P. aeruginosa biofilm induce membrane rigidification in P. aeruginosa.

**FIG 4:**
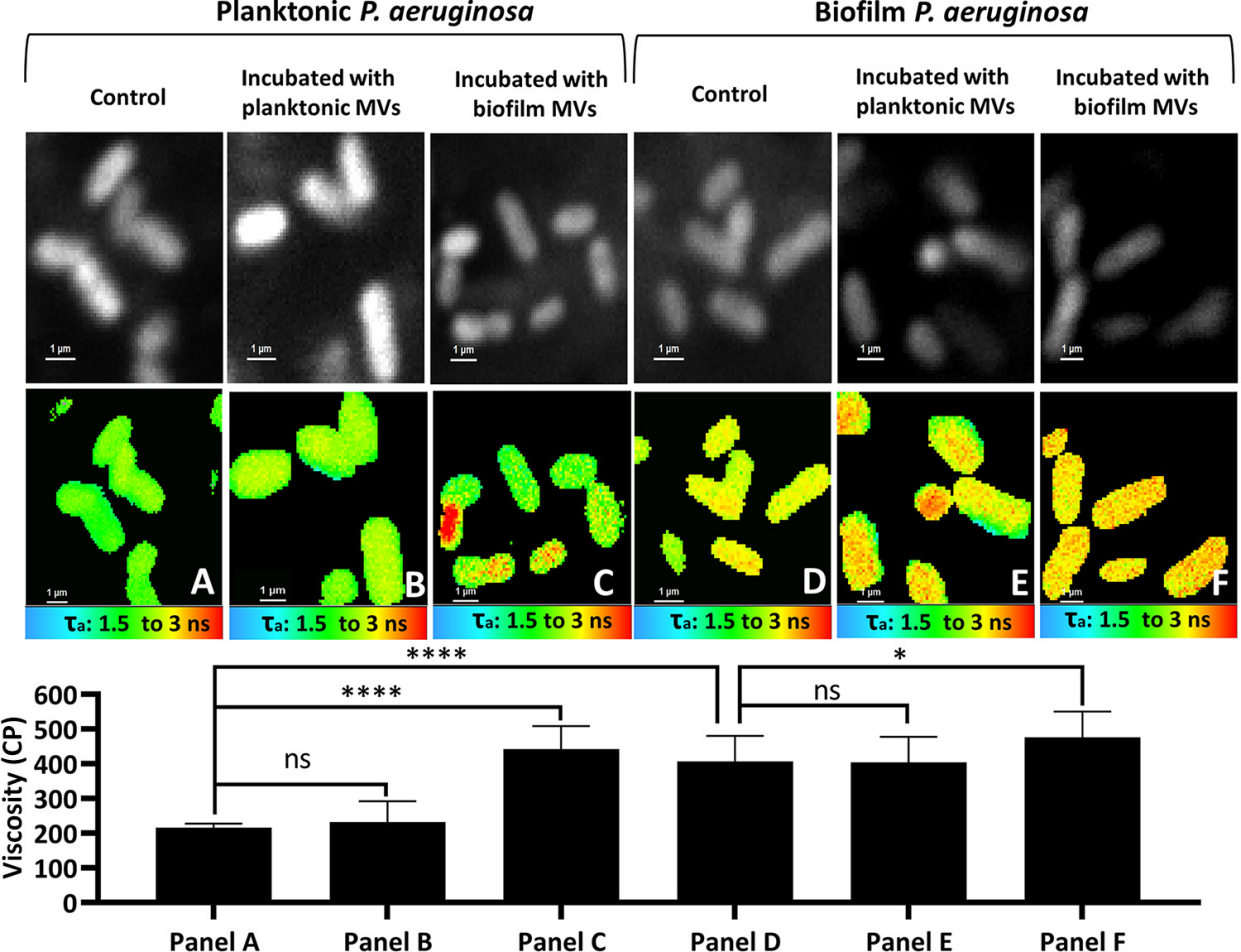
FLIM images and viscosity analysis. (Top) Intensity images. (Middle) Pseudo-colored FLIM images. Shown are planktonic control P. aeruginosa (A), planktonic P. aeruginosa incubated with MVs from the planktonic bacteria (B), planktonic P. aeruginosa incubated with MVs of the biofilm bacteria (C), control biofilm P. aeruginosa (D), the biofilm bacteria incubated with MVs of the planktonic bacteria (E), and biofilm P. aeruginosa incubated with MVs of the biofilm bacteria (F). (Bottom) The comparative viscosity analysis of the membranes according to the lifetime of BODIPY-C10. The data were obtained from two individual experiments, and in each experiment, at least 10 bacteria or three slides were considered to measure the lifetimes of the bacteria. The data are presented as means ± SD from independent replicates under each condition. Statistical analysis was performed by one-way ANOVA with Tukey’s multiple-comparison test. ****, *P* < 0.0001; *, *P* < 0.05; ns, not significant.

The two lifestyles of P. aeruginosa, i.e., planktonic and biofilm, result in remarkable differences in MV composition in terms of phospholipids (Fig. S3), mRNA (Fig. 2), and fatty acids (Fig. 3). We thus investigated (i) whether P. aeruginosa membranes are different in terms of their fluidity and (ii) whether the MVs produced by P. aeruginosa in the planktonic and biofilm growth modes interplay with bacteria and change their membrane fluidity. To answer these questions, first we compared the membrane fluidity of planktonic versus biofilm P. aeruginosa; second, we supplemented the bacterial culture media with MVs of planktonic and biofilm bacteria. The control groups were grown in media without supplementation of MVs. The fluidity of the bacterial membranes then was evaluated via FLIM (Fig. 4). The fluorescence lifetime (τ) of BODIPY-C10 correlates with the viscosity of the environment in which it is embedded. The longer lifetime of the probe indicates the higher viscosity of its microenvironment (35).

The FLIM analysis showed that the membrane of the planktonic P. aeruginosa is approximately two times less rigid (Fig. 4A) than that of the biofilm bacteria (Fig. 4D). Although no significant effect was observed on the membrane fluidity after incubation with MVs of the planktonic bacteria relative to their corresponding controls (Fig. 4B and E), MVs isolated from the biofilm bacteria significantly decreased the fluidity of both the planktonic and biofilm bacterial membrane compared with that of their controls (Fig. 4C and F). Indeed, incubating the planktonic bacteria with MVs of the biofilm bacteria rigidified the planktonic membrane to show a degree of fluidity similar to that of biofilm bacteria. Hence, we assume that the cross talk established via MVs of the biofilm bacteria, between biofilm bacteria and planktonic bacteria, and between bacteria inside the biofilm favors membrane rigidification.

### Decrease of membrane fluidity delays the growth of P. aeruginosa.

**FIG 5:**
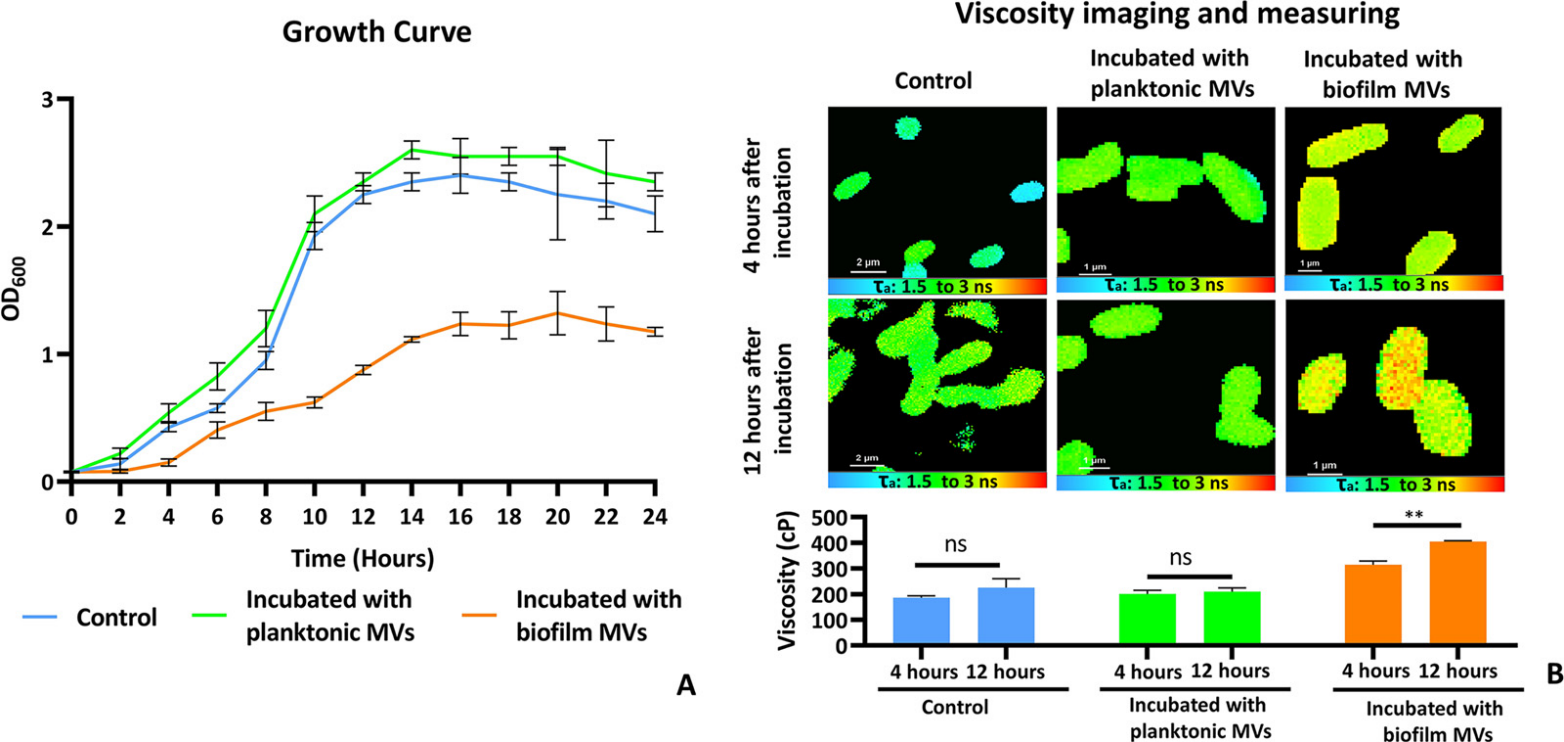
Growth curves of P. aeruginosa in the presence of MVs and the effect of incubation with MVs on membrane fluidity of P. aeruginosa. (A, left) Growth curves of the bacterial control culture (nonsupplemented medium), medium supplemented with MVs of the planktonic P. aeruginosa, and medium supplemented with MVs of the biofilm P. aeruginosa. (Top, right) FLIM images of planktonic P. aeruginosa in unsupplemented medium (control), medium supplemented with MVs of the planktonic bacteria, and medium supplemented with MVs of the biofilm bacteria after 4 h of incubation. (Middle) After 12 h of incubation. (Bottom) Calculated viscosity corresponding to the upper panels, according to the lifetime of BODIPY-C10. (B) The data were obtained from two individual experiments, and in each experiment, at least 10 bacteria or three slides were considered for measuring the lifetimes of the bacteria. The data are presented as means ± SD from independent replicates under each condition. Statistical analysis was performed by paired *t* test. **, *P* < 0.01; ns, not significant.

Comparison between the growth curves of P. aeruginosa in the presence of the MVs (from the planktonic and the biofilm bacteria) and in the unsupplemented medium (control) showed that supplementation of the media with MVs from biofilm bacteria negatively affected the bacterial growth (Fig. 5A). Two time points were selected to investigate the changes in membrane fluidity during bacterial growth: early logarithmic phase (4 h) and the end of the logarithmic growth phase (12 h). The FLIM study was performed on the bacteria. Results showed no significant difference between 4 and 12 h concerning the viscosity of the bacterial membrane in both control bacteria and bacteria treated with the MVs of the planktonic bacteria. In contrast, the viscosity of the bacterial membrane of bacteria grown in the presence of the MVs of biofilm bacteria significantly increased after 12 h of incubation compared with that after 4 h of incubation (Fig. 5B).

### The MV-driven cross talk between the planktonic and biofilm P. aeruginosa favors biofilm development.

**FIG 6:**
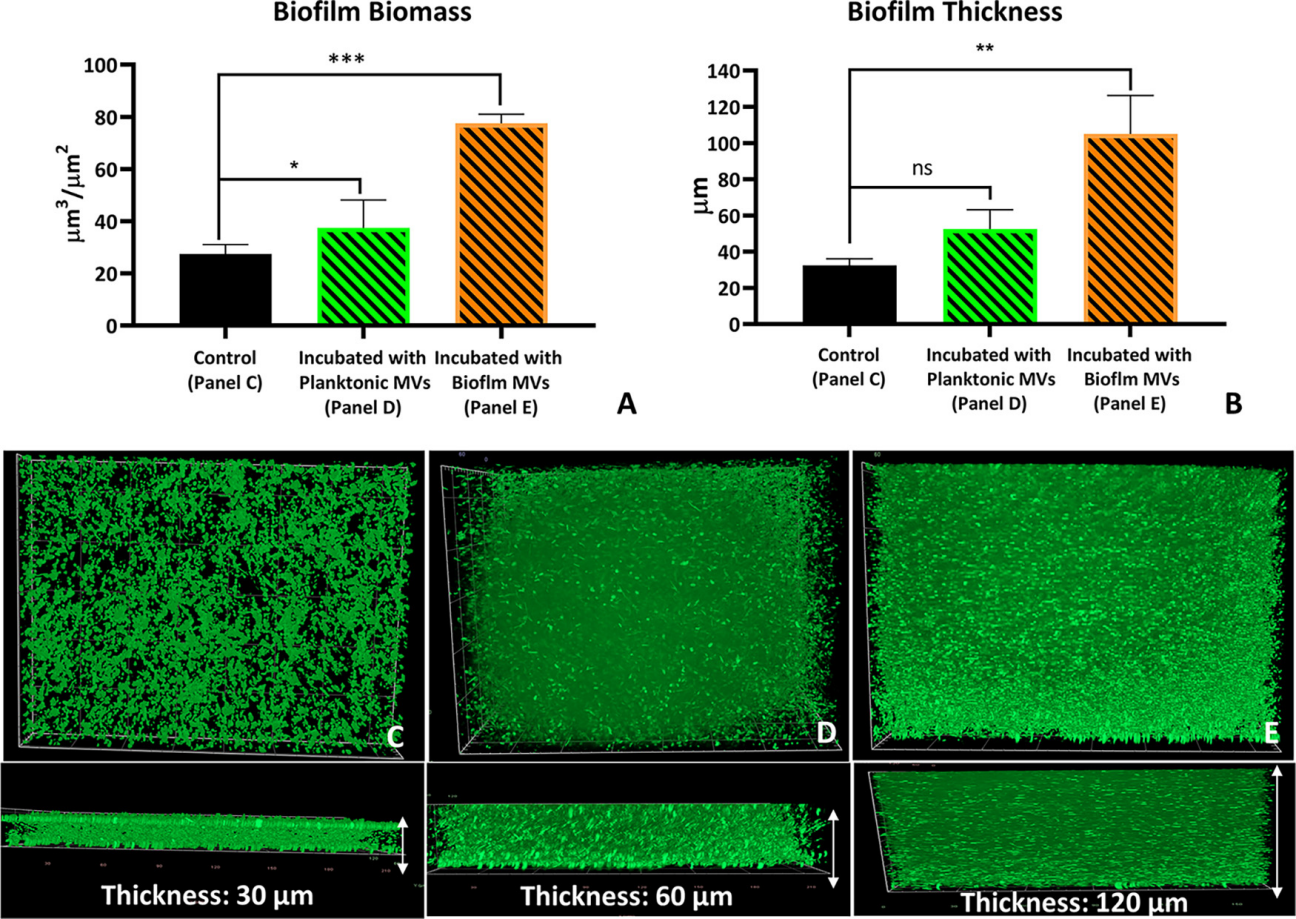
Effect of MVs on biofilm development in P. aeruginosa. (A, top) Biofilm biomass. (B, top) Biofilm thickness. (C, middle) The 3D biofilm architecture (stained with SYTO 9) relative to the control biofilm. (D) The biofilm incubated with MVs of the planktonic bacteria. (E) The biofilm incubated with MVs of the biofilm bacteria. (Bottom) The thickness of the biofilms corresponding to the upper panels. For each condition, three slides were prepared. The data are presented as means ± SD from three independent experiments. One-way ANOVA was performed for statistical analysis with Tukey’s multiple-comparison test. ***, *P* < 0.001; **, *P* < 0.01; *, *P* < 0.05; ns, not significant.

To further study the effect of the cross talk established between bacteria via their MVs, we investigated the effect of MVs on biofilm development (Fig. 6).

We observed that the incubation of the biofilm with MVs from the biofilm enhanced the biofilm biomass (Fig. 6A) and thickness (Fig. 6B) compared with that in the control group. Further, incubation with MVs of biofilm P. aeruginosa increased the biofilm biomass and thickness by more than 2 times and 4 times, respectively, than with the control. In contrast, the effects of MVs from the planktonic P. aeruginosa were weaker than those of MVs of the biofilm bacteria. The three-dimensional (3D) images of the biofilms (Fig. 6C to E) also show the difference between the thickness and biomass distribution of the biofilms due to the effects of MVs.

## DISCUSSION

This study revealed that the membrane viscosity of P. aeruginosa undergoes a dramatic increase when the planktonic bacteria shift to the biofilm lifestyle. Membrane rigidification endows the bacteria with the ability to survive the static lifestyle of biofilm (36). The differential enrichment of the membranes with phospholipid and distinct regulation of genes involved in lipid biosynthesis in the planktonic versus the biofilm bacteria is consistent with the findings of this study (2, 37). Interestingly, we observed that MVs could establish cross talk between the biofilm and the planktonic bacteria, favoring membrane rigidification and enhancing biofilm development.

Stressors inside the biofilm induce bacterial membrane lipid alterations that diminish membrane fluidity (38). Enriching the membrane with saturated fatty acids is a long-lasting strategy by which bacteria increase their membrane viscosity (2). Our result also confirmed that, compared with the membranes of biofilm P. aeruginosa, membranes of the planktonic bacteria are composed of a lower proportion of phospholipids with saturated fatty acids, which is in line with previous studies (6, 39). Phospholipids with distinct polar heads affect membrane fluidity, i.e., PG contributes to increased membrane rigidity (14) while CL decreases it (1). In this study, we observed a slight but significant increase of PG in the membrane of biofilm P. aeruginosa compared with that in the planktonic bacteria. The relative proportion of CL also was significantly higher in the membrane of the planktonic bacteria. Overall, these membrane modifications reduce bacterial cell exchanges in stressful environments such as during exposure to antibiotics or dwelling in biofilm (40).

Given that the *de novo* synthesis of the lipids is highly energy-demanding, P. aeruginosa is more likely to utilize preexisting lipids and lipids from external sources to change its lipid composition. Former studies revealed that P. aeruginosa mutants for long-chain fatty acids synthesizing enzymes (FadD1 and FadD2) could not utilize their host's nutrient source and form biofilms (41). Hence, using exogenous fatty acids is a strategy by which P. aeruginosa forms biofilms (42). FadD1 and FadD2 activate the exogenous and preexisting fatty acids for entering the cells’ lipid biosynthesis pathways (12). In P. aeruginosa, FadD1 and FadD2 have affinities with long- and medium-chain fatty acids; thus, C_16_ and C_18_ fatty acids might be precursors for these enzymes to form rigid membranes (42). In this study, it was observed that there are significantly higher transcripts of FadD1 and FadD2 in the biofilm P. aeruginosa than in the planktonic ones. Given that biofilm P. aeruginosa organisms have a less fluid membrane, the overexpression of these enzymes in the biofilm bacteria is in line with the effect of the corresponding enzyme in increasing the level of long-chain fatty acids, resulting in decreased membrane fluidity.

Additionally, in P. aeruginosa, DesA and DesB enzymes bypass *de novo* synthesis of unsaturated fatty acids because they oxidize existing saturated fatty acids (1, 8, 43). In this experiment, in biofilm P. aeruginosa, the relative expression of the two desaturases was significantly lower than that of the planktonic bacteria. Thus, shifting to a biofilm lifestyle could have a modulatory effect on the expression level of the desaturases. It is worth mentioning that although this study concentrated on the fluidity of the bacterial inner membrane (see Fig. S4 in the supplemental material), Gram-negative bacteria also undergo changes to the fluidity of their outer membrane to resist stressors. Changes in outer membrane fluidity occur because of alterations in the composition of lipid A and the types and concentrations of outer membrane proteins (5, 29).

10.1128/msphere.00187-22.4 FIG S4 FLIM images and lifetime measurements of bacterial cells and the spheroplasts. (A, upper) Intensity image. (Bottom) Pseudo-colored FLIM image; arrows indicate the planktonic P. aeruginosa and the spheroplast considered for calculating the probe’s lifetime. (B) Measurements of the probe’s lifetime in the membrane of the bacteria and the spheroplast. Download FIG S4, TIF file, 1.1 MB. Copyright © 2022 Mozaheb et al. 2022 Mozaheb et al. https://creativecommons.org/licenses/by/4.0/ This content is distributed under the terms of the Creative Commons Attribution 4.0 International license.

Our observation regarding the high abundance of lipid-enriched MVs produced by biofilm P. aeruginosa motivated us to study the role of MVs in altering membrane fluidity. We aimed to investigate whether the MVs affect membrane fluidity of the bystander bacteria and, more specifically, potential cross talk between the planktonic and the biofilm communities. The diversity in the origins of MVs offers them the capability of carrying various molecules (44). In particular, Turnbull et al. pointed out the explosive cell death phenomenon as an essential source of MVs in the biofilm of P. aeruginosa (22). This type of MV carries a part of the cytoplasm and inner membrane (cytoplasmic membrane), the so-called outer-inner membrane vesicles (or OIMVs) (45). We observed that MVs of the biofilm bacteria are significantly more associated with ATP, which is exclusively a cytoplasmic component. Thus, the larger amount of ATP associated with MVs of the biofilm bacteria indicates that a larger number of MVs have cytoplasmic material among the MVs of the biofilm bacteria than the MVs of the planktonic bacteria. This finding is in line with TEM observations regarding the higher presence of double-layer MVs (OIMVs).

We observed that saturated fatty acids are highly abundant in the MVs. A previous study by Tashiro et al. on the MVs of the planktonic P. aeruginosa at the late stationary phase of growth showed that MVs are enriched with PG and saturated fatty acids (14). Thus, it has been assumed that MVs of planktonic P. aeruginosa form by bulging of the rigid regions of the membrane (27).

This study revealed that the MVs of the biofilm bacteria, but not those of the planktonic bacteria, confer membrane rigidity to the planktonic bacteria. The induced rigidification via cross talk between biofilm and the planktonic P. aeruginosa could be due to their MV lipid compositions. In a complementary experiment, we extracted the total lipids of the MVs of planktonic and biofilm P. aeruginosa. We supplemented the growth media of the planktonic bacteria with lipid extracts, and we investigated the bacterial membrane viscosity. This experiment showed roughly the same trend of effect compared to the investigation related to the effect of MVs (Fig. S5). The MVs are composed of saturated long-chain fatty acids (C_16:0_ and C_18:0_) that are significantly abundant in the MVs of the biofilm bacteria. Presumably, these fatty acids can serve as an external source of lipids (2, 43, 46).

10.1128/msphere.00187-22.5 FIG S5 FLIM images and viscosity analysis. (Top) Pseudo-colored FLIM images. Planktonic control P. aeruginosa (A), planktonic P. aeruginosa incubated with the total lipids extracted from the planktonic MVs (B), and planktonic P. aeruginosa incubated with the total lipids extracted from the MVs of the biofilm bacteria (C). (Middle) The lifetime analysis of BODIPY-C10 corresponding to the upper panels. (Bottom) The comparative viscosity analysis of the membranes according to the probe's lifetime. The data were obtained from individual observations, and they are presented as means ± SD from independent replicates under each condition. Statistical analysis was performed by one-way ANOVA with Tukey’s multiple comparison test. **, *P* < 0.01; *, *P* < 0.05. Download FIG S5, TIF file, 0.9 MB. Copyright © 2022 Mozaheb et al. 2022 Mozaheb et al. https://creativecommons.org/licenses/by/4.0/ This content is distributed under the terms of the Creative Commons Attribution 4.0 International license.

Given that in response to the environmental stress P. aeruginosa benefits from decreased membrane fluidity (38), the capability of membrane rigidification is likely to be dispersed in the bacterial community and transferred to other bacteria. This study showed that the MVs of biofilm P. aeruginosa are more associated with the cytoplasmic components than the MVs of the planktonic bacteria. Hence, MVs of the biofilm bacteria can carry transcripts and enzymes contributing to the membrane rigidification and transfer them to other bacteria in the population. Further studies are required to show the delivery of these cargos to the recipient cells.

Very interestingly, investigation of the effect of the MVs on P. aeruginosa growth rate and fluidity of the bacterial membrane indicated that MVs of the biofilm P. aeruginosa dictate membrane rigidification. Given that the fluid status of the bacterial membrane ensures the passage of nutrients to the cytoplasm, a decrease in the bacterial rate of growth seems to be related to the reduction of membrane fluidity and diminished capability of nutrient acquisition (33).

This study noted that in the biofilm, P. aeruginosa presents a rigid membrane; MVs produced by biofilm bacteria increase the membrane rigidity in both planktonic and biofilm P. aeruginosa. Therefore, we were interested in examining MV-induced biofilm development. We found that, compared with control bacteria, and on incubating with MVs of planktonic P. aeruginosa, biofilm thickness and biomass increased significantly due to the incubation of the biofilm with MVs of the biofilm bacteria.

Biofilm formation is aided by reducing bacterial membrane fluidity (30). As mentioned above, produced by biofilm bacteria, MVs provide an exogenous source of saturated fatty acids for the bacteria, resulting in increasing propensity to form a biofilm population (17). Additionally, nutrient depletion resulting from membrane rigidification triggers the stress response pathways in the cells, resulting in activation of extracellular polymeric substance (EPS) matrix production (47, 48). Moreover, PQS carried by MVs (planktonic and biofilm) promotes the formation of cell aggregates and biofilm phenotypes (49), and the presence of eDNA packed inside the MVs (50) could explain the increase of the biofilm biomass due to incubation with the MVs. Membrane alteration, via interplay between the planktonic P. aeruginosa and the biofilm bacteria, mediated by MVs, can alter phenotype-related pathogenic characteristics such as the ability of bacteria to form biofilms (2, 4).

In this study, the use of BODIPY-C10 probe was combined with a microscopy approach (FLIM) to compare the fluidity of planktonic and biofilm P. aeruginosa. We found that decreased membrane fluidity could induce biofilm development capability. MVs are among the virulence factors of P. aeruginosa; thus, their production not only favors the producing bacteria but is also beneficial for the bystander bacteria. Switching from planktonic to biofilm mode of growth is accompanied by variations in MV composition. MVs maintain interplay between these two lifestyles by establishing a cross talk between the biofilm and the planktonic bacteria. Our study suggested that the cross talk mediated by MVs of the biofilm bacteria favors membrane rigidification. Biofilm is associated with poor outcomes of infections caused by P. aeruginosa. Hence, gaining a mechanistic view of the various physiological changes that the bacterium goes through to shift from the planktonic lifestyle to the biofilm lifestyle is very important for identifying a practical way to prevent bacteria from establishing this lifestyle. These observations show that the bacterial cross talk mediated by MVs can be a driving force for membrane rigidification and biofilm formation. This study helps elucidate this critical pathogen's pathophysiology to pave the way for designing new therapeutic approaches.

## MATERIALS AND METHODS

### Bacterial cultivation and MV isolation. (i) Planktonic culture.

Overnight culture of the P. aeruginosa PAO1 strain was suspended in LB (Miller’s modification) at a concentration of 10^5^ CFU/mL; the pH was adjusted to 7.2 using NaOH. The bacteria were incubated for 20 h at 37°C with shaking at 180 rpm.

### (ii) Biofilm culture.

The bacteria were inoculated to LB at a final concentration of 10^7^ CFU/mL, and the biofilms were formed in 24-well polystyrene plates and incubated at 37°C without shaking for 20 h. The media then were discarded, and the adhering biofilms were washed three times with PBS to remove the remaining planktonic bacteria. The biofilms were then harvested.

### (iii) MV isolation, purification, and characterization.

For isolation of MVs from the planktonic bacteria, bacterial cells were grown in one-fifth volumes of Erlenmeyer flasks. After 12 h (at the end of the logarithmic growth phase), the cells were removed by centrifugation at 2,978 × *g* for 20 min at 4°C (Eppendorf 5810 R centrifuge, A-4-62 rotor). The supernatant was passed through a 0.45-μm polyvinyl difluoride (PVDF) filter (Whatman) and subjected to ultracentrifugation at 150,000 × *g* for 3 h at 4°C (80 Ti rotor; Beckman). The supernatant was discarded, and the pellet was resuspended in 10 mM HEPES–0.85% NaCl buffer, pH 7.2 (MV buffer) (49). The isolation of the MVs from biofilm bacteria was done according to the protocol utilized by Schooling and Beveridge (17). Isolated MVs were further purified via gradient density of OptiPrep-iodixanol (Sigma-Aldrich) in MV buffer, as described previously (51).

MV size analysis was performed using ZetaVIEW S/N 18-400. Lipid quantification was conducted using FM 4-64 dye, and the method was adapted from Hirayama et al. (52). FM 4-64 is an amphiphilic molecule that is fluorescent in a lipophilic environment. The fluorescence intensity of this probe increases as a function of enhancement in lipid concentration of the probe’s environment.

Briefly, the samples were mixed with the dye (at 5 μg/mL), and the mixture was put in a transparent 96-well plate and incubated at room temperature for 30 min in dim light. The dye was excited at 535 nm, and the emission was read at 625 nm (SpectraMax M3). A calibration curve was built using increasing concentrations of water-soluble linoleic acid (L5900; Sigma-Aldrich) and 5 μg/mL FM 4-64 dye.

### (iv) Incubation of the bacteria with the MVs in planktonic culture.

The growth media of the bacterial culture in the middle of the logarithmic phase of growth (10^7^ CFU/mL) were supplemented with MVs at a final concentration of 10^8^ particles/mL. To exclude the effect of eDNA associated with the surfaces of the MVs, before adding them to the media, the MVs were incubated with DNase (TURBO DNA-free kit). After 8 h, the bacterial cells were harvested and washed twice with PBS for the FLIM experiment.

### (v) Incubation of the bacteria with the MVs in biofilm culture.

The biofilm culture was supplemented with the MVs (10^8^ particles/mL) within 12 h of biofilm formation (i.e., the attachment of the cells to the wells). The cultures were incubated for 8 h, and then the fluidity of their membranes was compared with that of the control cells (i.e., bacteria not treated with the MVs). Further, to prepare the bacterial growth curves in the presence of MVs, the bacteria (10^5^ CFU/mL) inoculated in the growth media were enriched with MVs or left untreated. The optical density of the culture medium at 600 nm was measured every 2 h.

### Negative staining and TEM.

To study the morphology of the MVs via transmission electron microscopy (TEM), a 5-nm carbon layer was electron-beamed on top of the Formvar-coated copper grids (300 mesh), and then 3.5 μL of the isolated MVs (diluted samples) was put on the grids for 5 min. The samples were glow-discharged in a Leica ACE600 coating machine (Leica, Vienna, Austria). Next, 20 μL of 2% uranyl acetate (SPI Supplies) was added. After 5 min, the excess liquid was removed. The samples were observed and imaged in a JEOL 1400 transmission electron microscope equipped with an EMSIS Quemesa camera (11 Mpxl; EMSIS, Münster, Germany) at an accelerating voltage of 80 kV and a pixel size of 0.7 nm (20kX).

### ATP quantification of the MVs.

The concentration of ATP present in the MVs was measured using BacTiter-Glo microbial cell viability assay (Promega). The purified MVs isolated from planktonic and biofilm P. aeruginosa were adjusted to equal numbers of MVs (10^8^ particles), and they were subjected to ATP assay via the established protocol (53).

### Phospholipid and fatty acid determination of the membranes and the MVs.

Membrane isolation and phospholipid and fatty acid determination of the membranes and the MVs are provided in the supplemental material (Text S1).

10.1128/msphere.00187-22.10 TEXT S1 The procedures for the synthesis of BODIPY-C10, phospholipid and fatty acid composition analysis, and FLIM imaging and analysis. Download Text S1, DOCX file, 0.04 MB. Copyright © 2022 Mozaheb et al. 2022 Mozaheb et al. https://creativecommons.org/licenses/by/4.0/ This content is distributed under the terms of the Creative Commons Attribution 4.0 International license.

### FLIM imaging and analysis.

The procedure for synthesis of probe (BODIPY-C10), slide preparation steps, detail of FLIM imaging (Fig. S6), calculation, and analysis (Fig. S7 and S8) are presented in the supplemental material (Text S1).

10.1128/msphere.00187-22.6 FIG S6 Lifetime histograms. Images show the distribution of the lifetimes on the FLIM image of membrane of planktonic P. aeruginosa (A) and membrane of biofilm P. aeruginosa (B). The histograms had a Gaussian distribution (goodness of fit, *R*^2^ ≥ 0.95). Download FIG S6, TIF file, 1.0 MB. Copyright © 2022 Mozaheb et al. 2022 Mozaheb et al. https://creativecommons.org/licenses/by/4.0/ This content is distributed under the terms of the Creative Commons Attribution 4.0 International license.

10.1128/msphere.00187-22.7 FIG S7 Fluorescence intensity decay of the probe in the membrane of planktonic P. aeruginosa. The decay curve was analyzed via a biexponential model with the goodness of fit equal to 1.139. FLIM fit was acquired using SymPhoTime 64 software. Download FIG S7, TIF file, 1.3 MB. Copyright © 2022 Mozaheb et al. 2022 Mozaheb et al. https://creativecommons.org/licenses/by/4.0/ This content is distributed under the terms of the Creative Commons Attribution 4.0 International license.

### Biofilm preparation and imaging.

Preparation and supplementation of the biofilm were performed similarly to the protocols mentioned above in 24-well polystyrene plates with a coverslip at the bottom. After 20 h, the planktonic bacteria were removed, and the biofilms were washed with PBS and stained with SYTO 9 (a membrane-permeable fluorophore targeting double-stranded DNA). After 15 min, the leftover dye molecules were removed. After washing with PBS, the coverslips were mounted and visualized on a cell observation spinning disk microscope (Carl Zeiss) with an oil immersion 40× objective. SYTO 9 was detected in the green channel (excitation/emission, 488/502 to 538 nm), and the images were acquired at a resolution of 1,388 by 1,040 pixels in the Z-stacks scanning mode. The 3D images were obtained using ZEN 2.6 (blue edition) software. Further analyses of the biomass and biofilm thickness were done using COMSTAT 2.1.

### Quantitative PCR analysis.

The bacterial cells (~10^9^ CFU) were subjected to RNA extraction using an RNA extraction kit (INVITEK), and the extracted RNAs were treated with DNase (TURBO DNA-free kit); corresponding cDNA synthesis then was performed. qPCR was performed using SYBR green supermix (Bio-Rad) and specific primers for *fadD1*, *fadD2*, *desA*, and *desB* genes (Table S1). The relative levels of gene expression were determined via the ΔΔ*C_T_* method and normalized using 16S rRNA as a control (54). For comparing the transcript cargos of MVs, similar particle numbers of the purified MVs from planktonic and biofilm bacteria were subjected to RNA isolation via the above-mentioned method. The mRNA levels in MVs were determined relative to the detected corresponding fragments of the genes and/or mRNA in the nontranscribed RNA samples (RT−) via the ΔΔ*C_T_* method.

10.1128/msphere.00187-22.9 TABLE S1 Primer sequences. Download Table S1, DOCX file, 0.01 MB. Copyright © 2022 Mozaheb et al. 2022 Mozaheb et al. https://creativecommons.org/licenses/by/4.0/ This content is distributed under the terms of the Creative Commons Attribution 4.0 International license.

### Data availability.

All the information related to this study and data generated during this study are included in this published article and its supplemental material.

10.1128/msphere.00187-22.8 FIG S8 Lifetime analysis of BODIPY-C10 incorporated into the membrane of P. aeruginosa under various conditions. Top panels are intensity images. Middle panels are pseudo-colored FLIM images showing the lifetime maps of the probe over the surface of the bacteria (A to F). The bottom panel shows the lifetime analysis of the probe corresponding to the upper panels. The data are presented as means ± SD from independent replicates under each condition. Statistical analysis was performed by one-way ANOVA with Tukey’s multiple-comparison test. ****, *P* < 0.0001; *, *P* < 0.05; ns, not significant. Download FIG S8, TIF file, 1.5 MB. Copyright © 2022 Mozaheb et al. 2022 Mozaheb et al. https://creativecommons.org/licenses/by/4.0/ This content is distributed under the terms of the Creative Commons Attribution 4.0 International license.
