# Supplementary figures and images for: Contribution of Membrane Vesicle to Reprogramming of Bacterial Membrane Fluidity in Pseudomonas aeruginosa
Source: mSphere. 2022 May 23;7(3):e00187-22. doi: 10.1128/msphere.00187-22 (PMC9241526; doi:10.1128/msphere.00187-22)

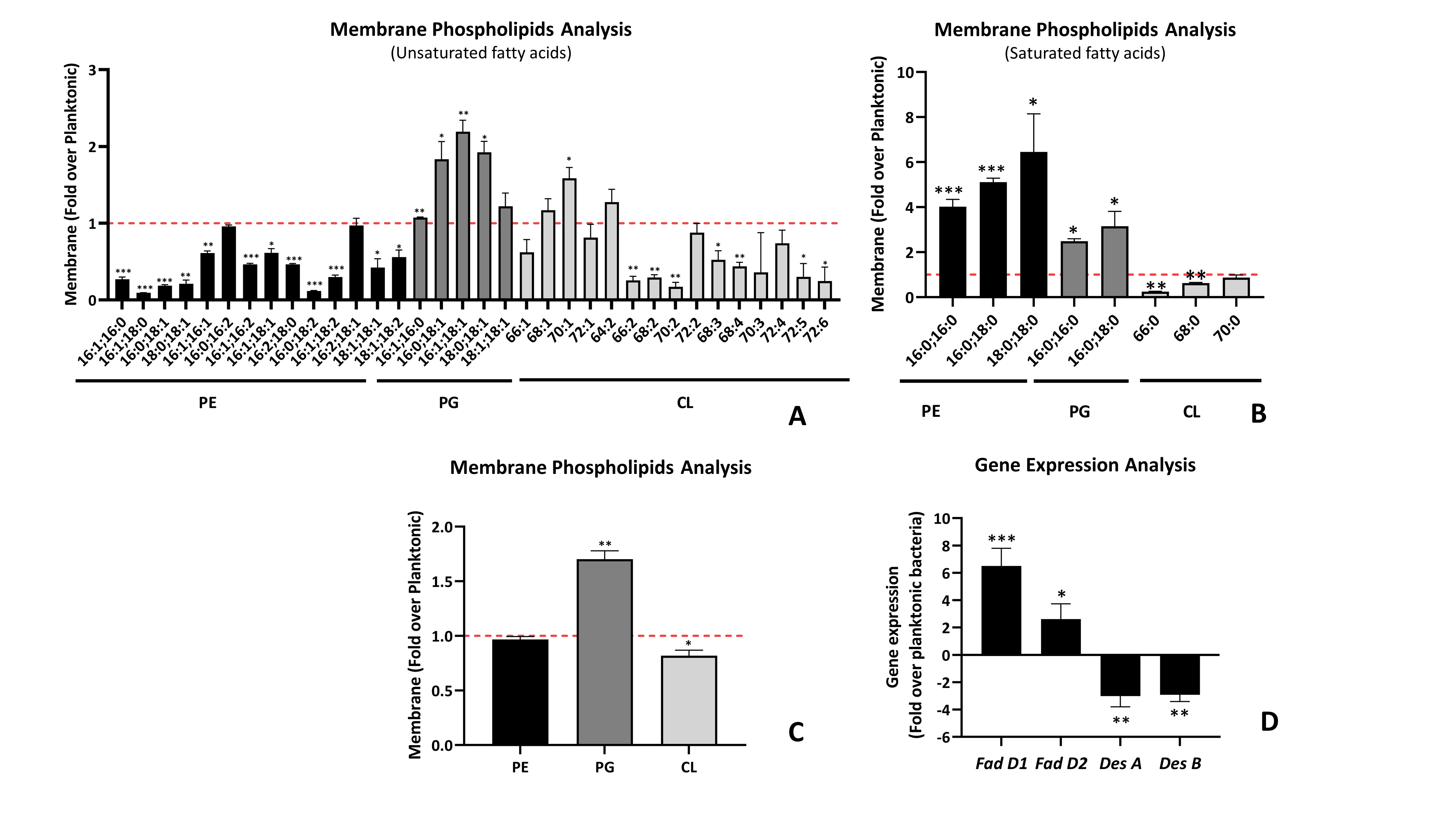

Supplement: FIG S1 [file msphere.00187-22-s0003.tif]

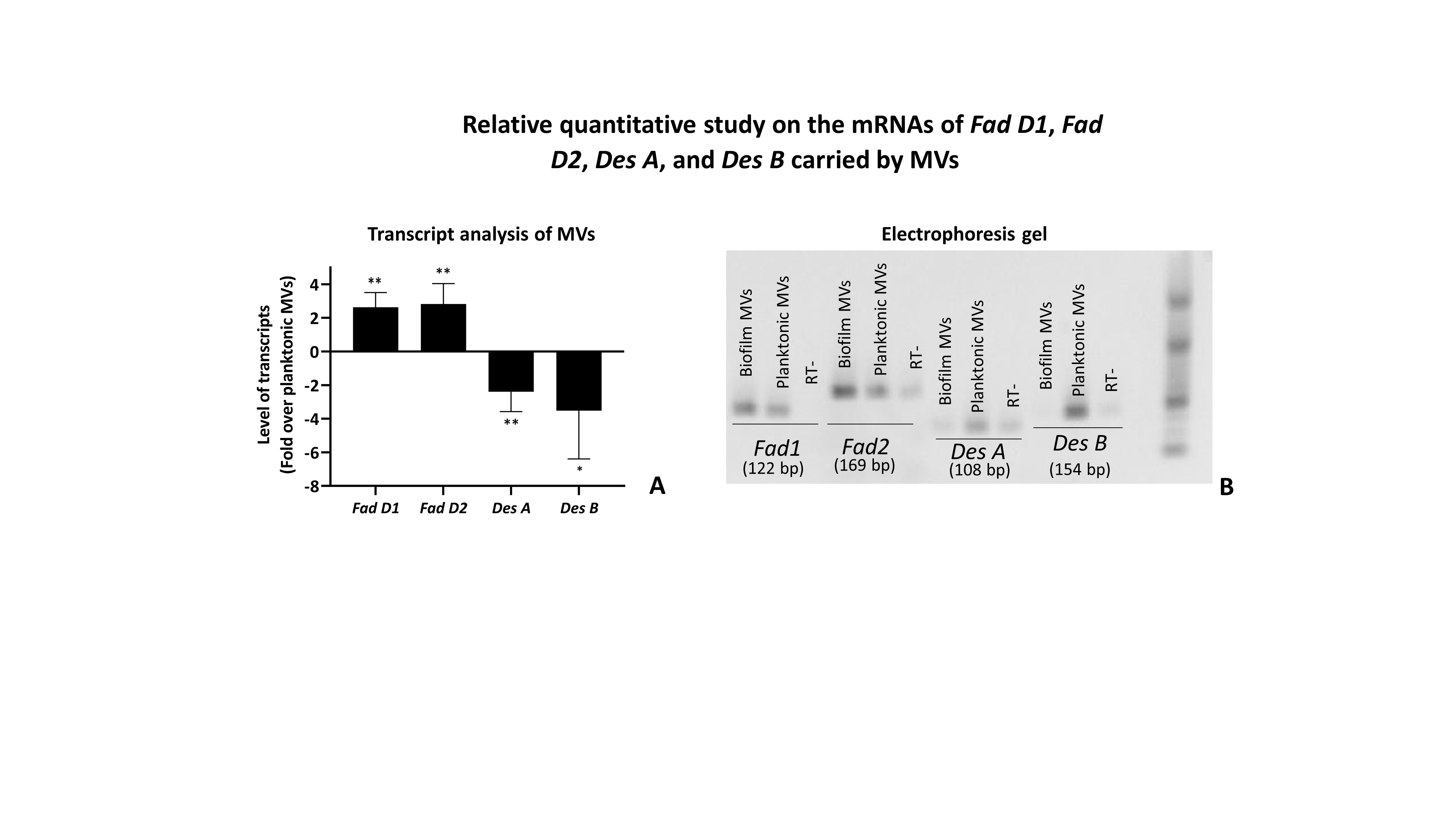

Supplement: FIG S2 [file msphere.00187-22-s0004.tif]

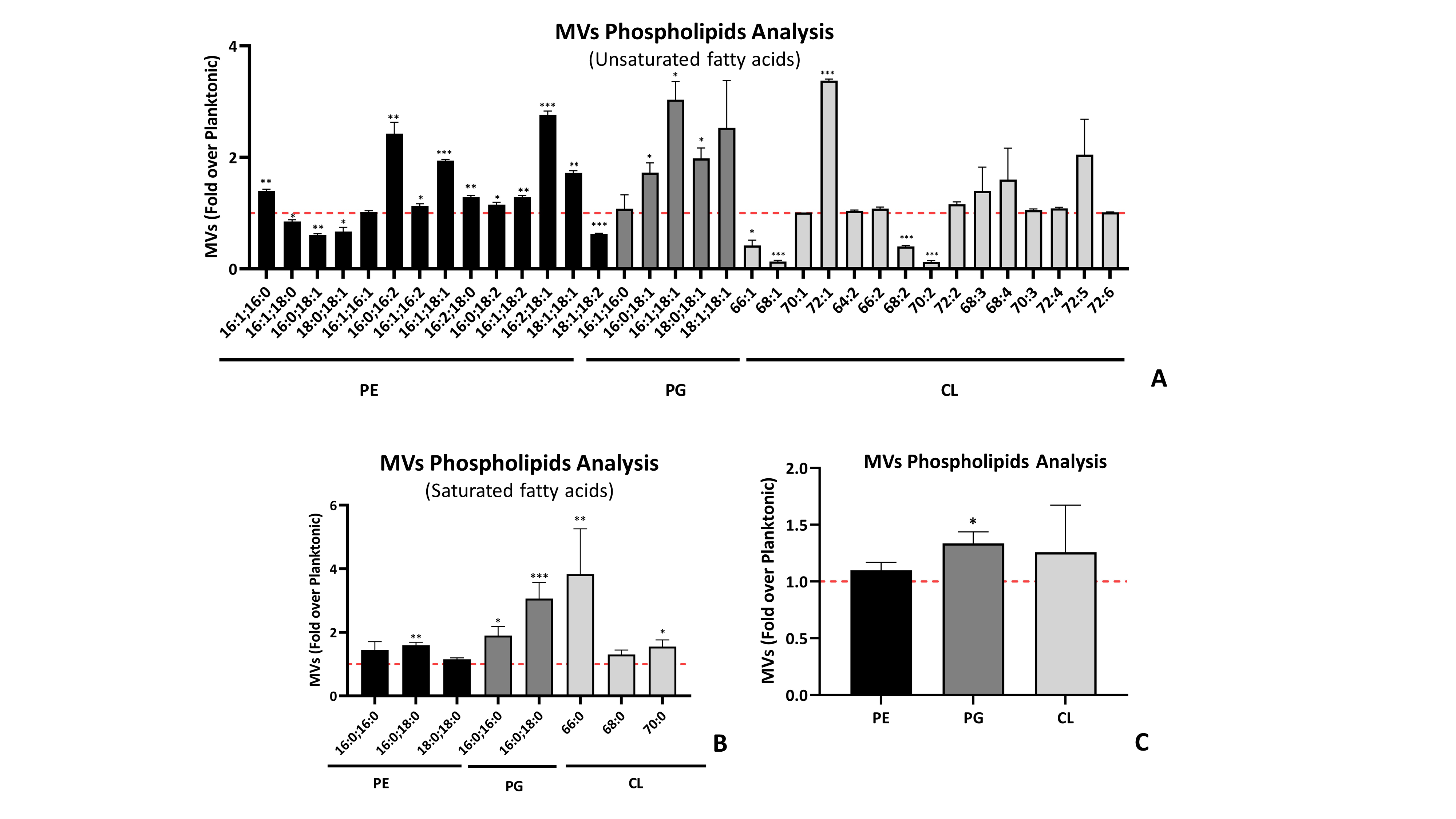

Supplement: FIG S3 [file msphere.00187-22-s0005.tif]

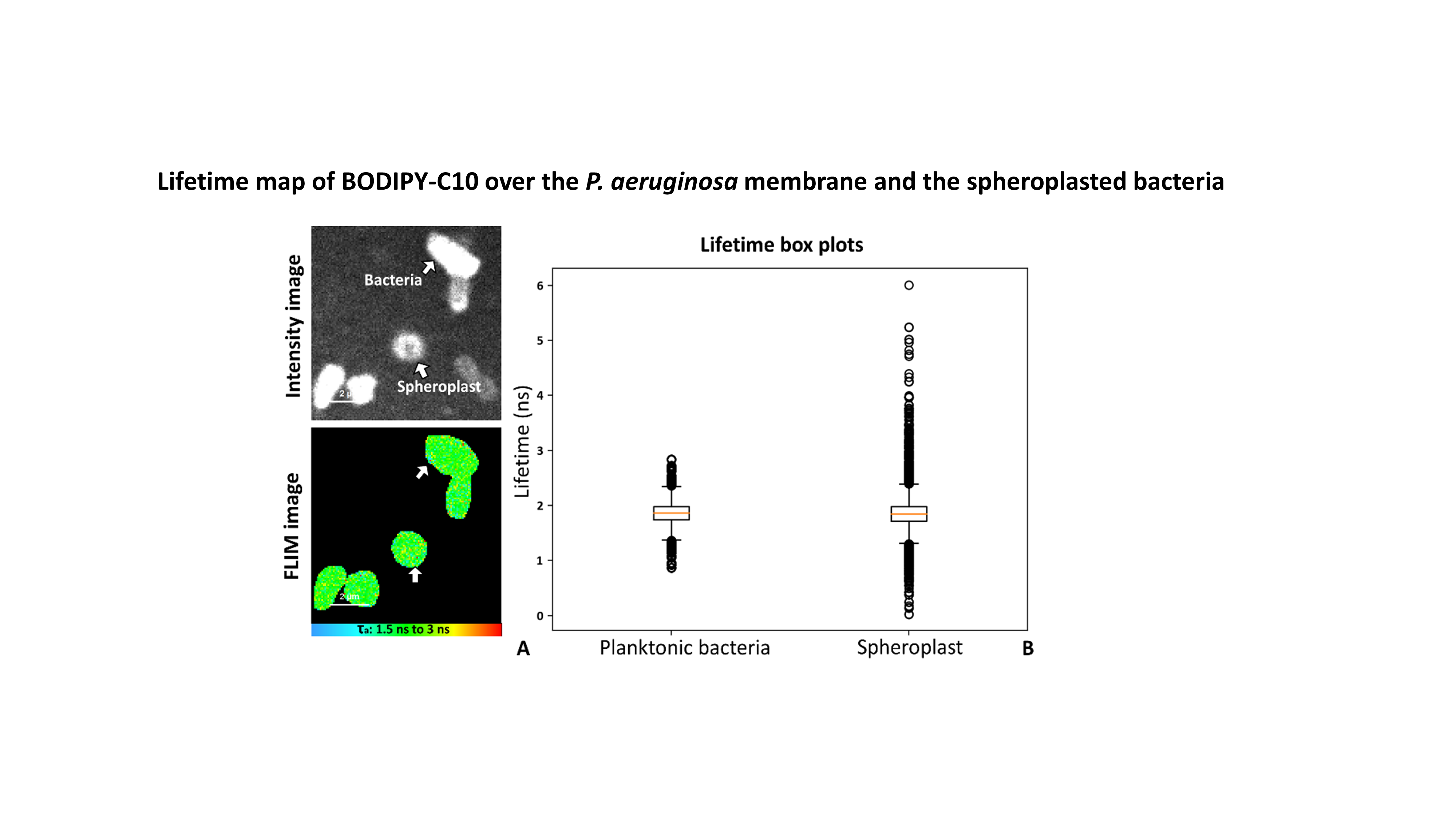

Supplement: FIG S4 [file msphere.00187-22-s0006.tif]

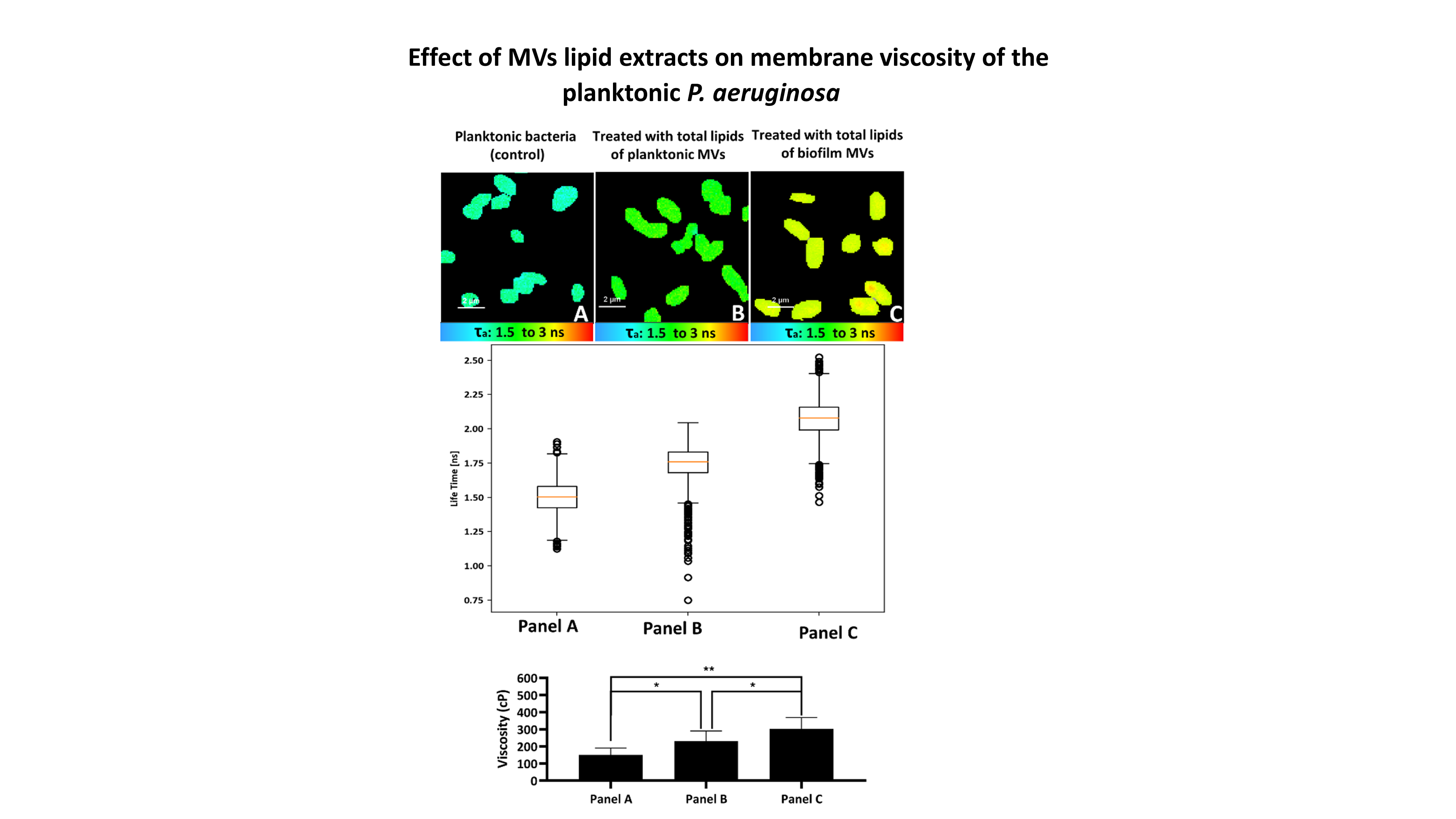

Supplement: FIG S5 [file msphere.00187-22-s0007.tif]

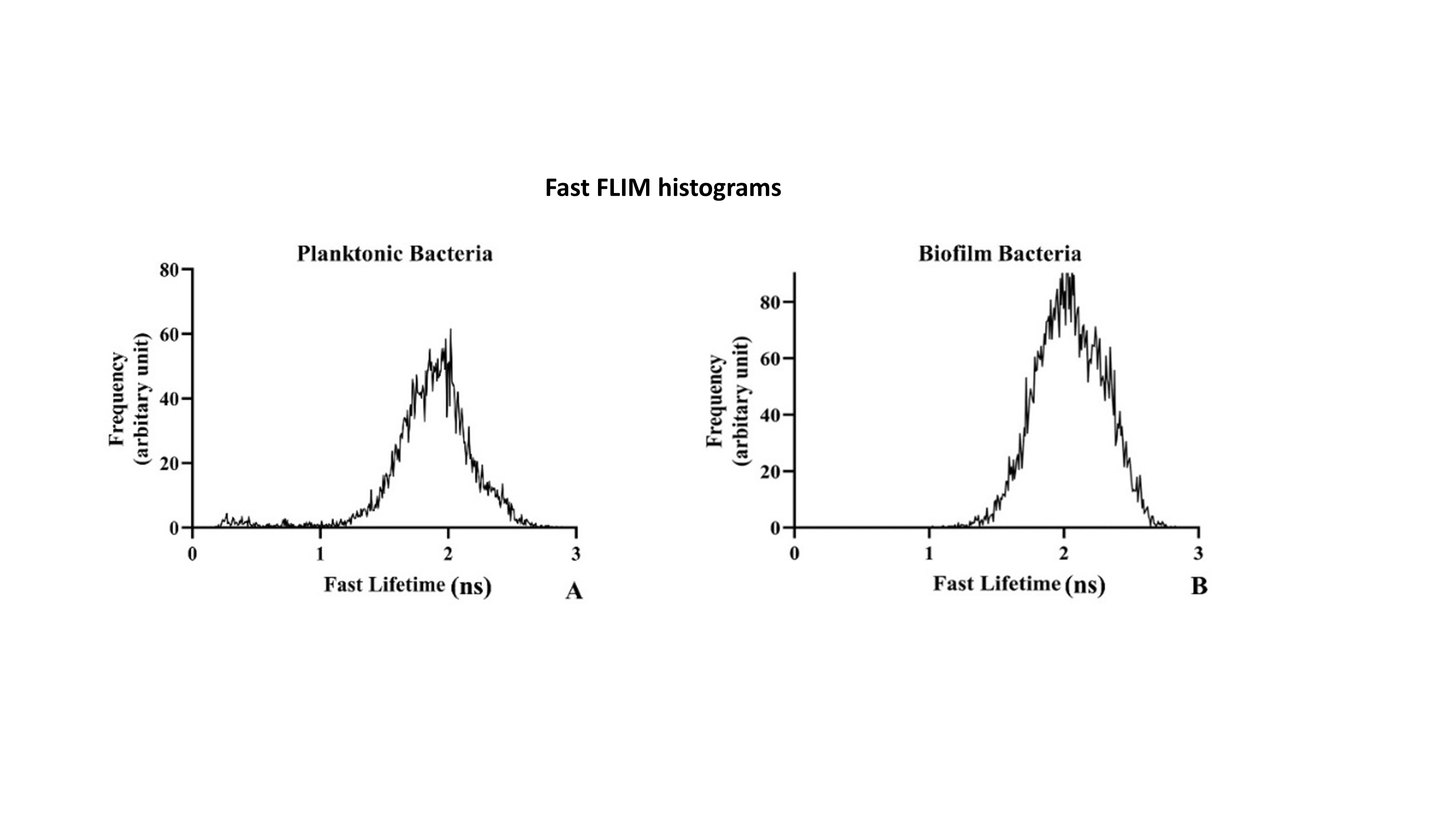

Supplement: FIG S6 [file msphere.00187-22-s0008.tif]

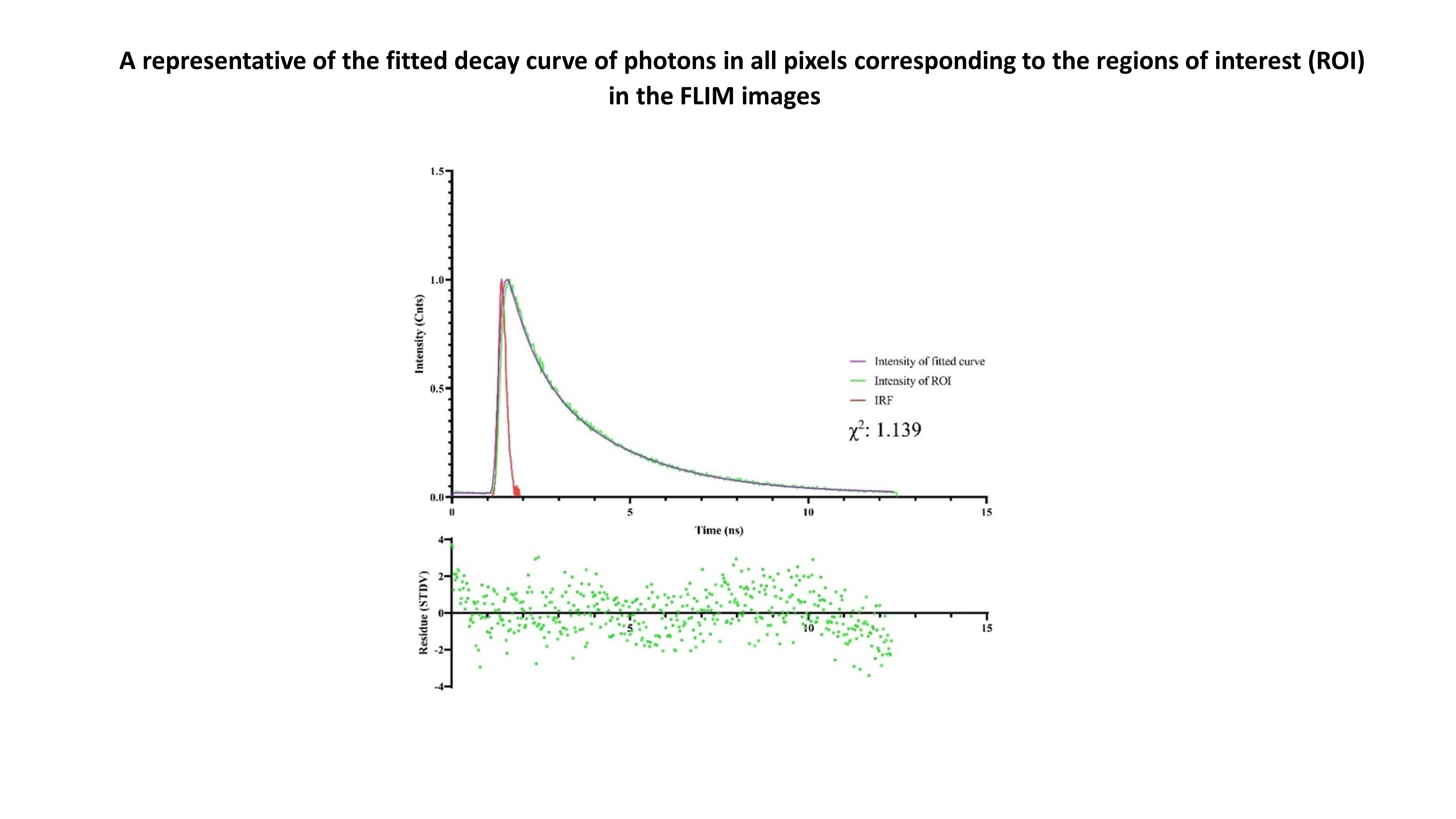

Supplement: FIG S7 [file msphere.00187-22-s0009.tif]

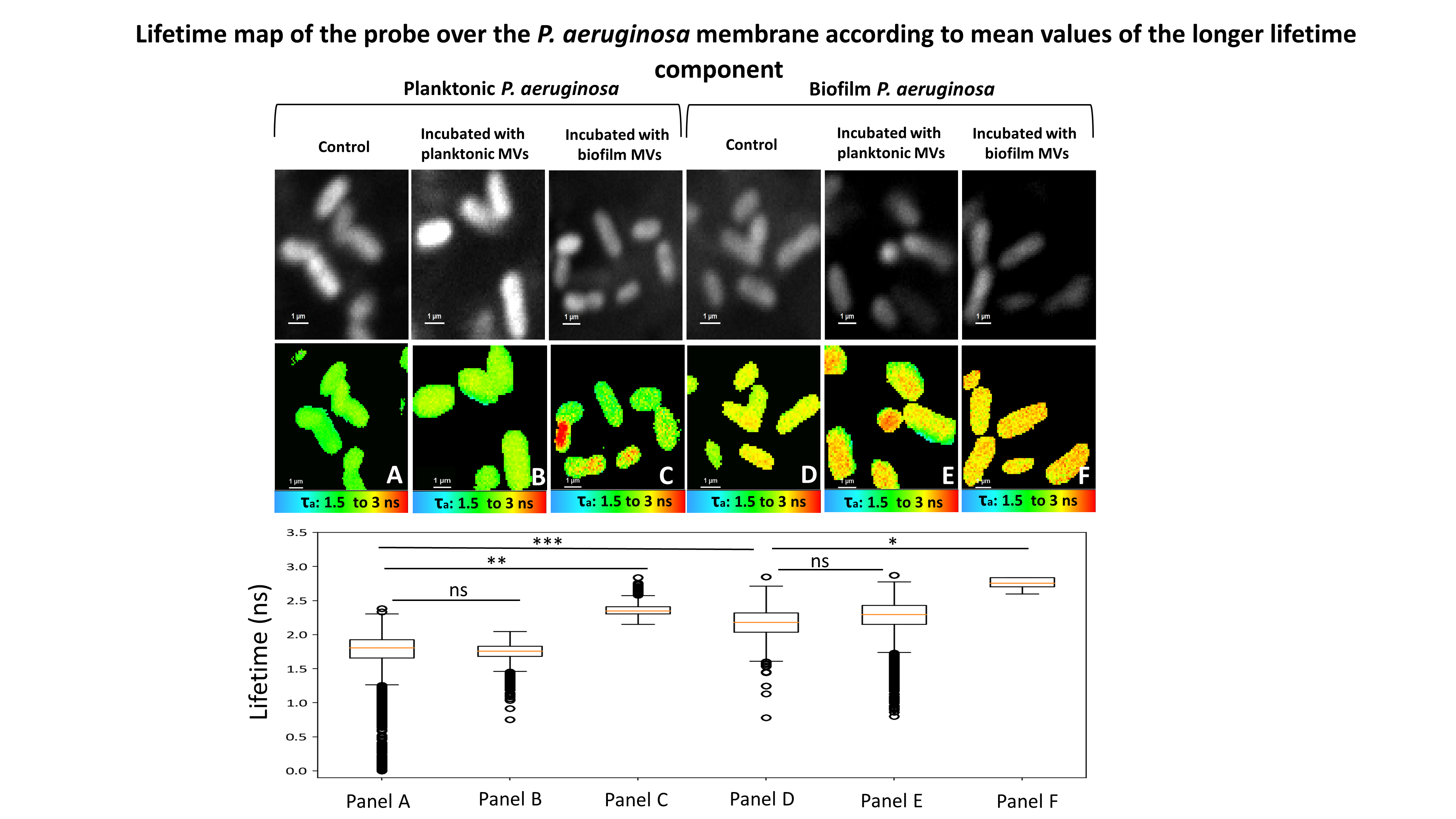

Supplement: FIG S8 [file msphere.00187-22-s0010.tif]
