# Supplementary material for: Contribution of Membrane Vesicle to Reprogramming of Bacterial Membrane Fluidity in Pseudomonas aeruginosa
Source: mSphere. 2022 May 23;7(3):e00187-22. doi: 10.1128/msphere.00187-22 (PMC9241526; doi:10.1128/msphere.00187-22)
Supplement: TEXT S1 [file msphere.00187-22-s0001.docx]

**Supplementary material**

**Contribution of membrane vesicle to reprogramming of bacterial membrane fluidity in *Pseudomonas aeruginosa***

Negar Mozaheb^1^, Patrick Van Der Smissen^2^, Tomas Opsomer^3^, Eric Mignolet^4^, Romano Terrasi^5^, Adrien Paquot^5^, Yvan Larondelle^4^, Wim Dehaen^3^, Giulio G. Muccioli^5^, Marie-Paule Mingeot-Leclercq^1*^.

1. Université catholique de Louvain, Louvain Drug Research Institute, Cellular & Molecular Pharmacology Unit (FACM), Brussels, Belgium.

2. Université catholique de Louvain, de Duve Institute, CELL Unit and PICT Platform, Brussels, Belgium.

3. KU Leuven, Department of Chemistry, Molecular Design and Synthesis, Celestijnenlaan 200F, 3001 Leuven, Belgium.

4. Université catholique de Louvain, Louvain Institute of Biomolecular Science and Technology, Louvain-la-Neuve, Belgium.

5. Université catholique de Louvain, Louvain Drug Research Institute, Bioanalysis and Pharmacology of Bioactive Lipids Research Group , Brussels, Belgium.

**The procedure for the synthesis of BODIPY-C10 and slide preparation steps for FLIM**

*BODIPY-C10 preparation*: The viscosity-sensitive fluorophore 8-(4-(decyloxy)phenyl)-4,4-difluoro-4-bora-3a,4a-diaza-*s*-indacene (BODIPY-C10) was synthesized according to previously described methods (1-3). Briefly, the intermediate dipyrromethane was prepared from 4-(decyloxy)benzaldehyde (1) (472 mg, 1.8 mmol) *via* a procedure reported for the synthesis of 8-(4-((10-iododecyl)oxy)phenyl)-BODIPY (3). Next, oxidation and complex formation with boron trifluoride (3 h) were carried out as described by Kuimova *et al.* (2), which gave a BODIPY-C10 yield of 37 % over 3 steps (281 mg, 0.66 mmol).

*Slide preparation*: The bacterial slide preparation protocol was adapted from a previous study (4), with some modifications. Briefly, cells at the concentration of 10^7^ CFU/mL were resuspended in the PBS containing BODIPY-10 (0.5 mM) supplemented with 0.1% w/v of glucose. The cells were labeled by mixing and incubating at 37°C with shaking. Subsequently, 200 µL of the cell suspensions were immobilized on the 8-well Ibidi chamber slide (IBIDI™ GmbH, Germany), which was precoated with 0.1% poly-L-Lysin.

**Lipid composition analysis**

*Isolation of the bacterial membranes*: The outer and inner membranes of the bacteria were isolated according to the protocol established by Contreras et al., with modifications (5). Briefly, the bacterial pellets were prepared according to the protocol mentioned for bacterial culture preparation. Then, a pellet of 10^9^ CFU of bacteria was obtained by centrifugation at 2978 g for 20 minutes at 4°C (Eppendorf 5810 R centrifuge; A-4-62 rotor). The pellet was resuspended in a buffer containing 1 M sucrose, 10 mM Tris-HCl, and 150 μg/mL lysozyme. The suspension was kept on ice for 5 minutes, and then EDTA solution (1.5 M, pH: 7.5) was slowly added to the mixture. The resulting spheroplasts were exposed to ultrasonic waves at 40 Hz frequency for 45 minutes at room temperature, and then the intact cells were pelleted down via centrifugation (2978 g for 20 minutes at 4°C (Eppendorf 5810 R centrifuge; A-4-62 rotor)). The supernatant was subjected to ultracentrifugation for 38473 g (rotor: Beckman 80Ti) for 90 minutes. The pellet contains the inner membrane of *P. aeruginosa*.

*Lipid analysis*: The lipid content of the different samples was analyzed by LC-MS. Briefly, lipids from the membranes and from the MVs were analyzed after liquid/liquid extraction (CH_2_Cl_2_-CH_3_OH-H_2_O, 4:2:1, v/v/v/) under acidic condition in the presence of internal standards (17:0-LPC, 17:1-LPE, 17:1-LPG, and 14:0/14:0/14:0/14:0-Cardiolipin). Phospholipid analysis was performed using a Xevo-TQS (from Waters), and cardiolipin analysis was carried out on an LTQ-orbitrap (from Thermo Fisher Scientific).

For the phospholipids, an HSS LC-18 column 100×2.1mm, 1.8µm (Waters) at a temperature of 40°C was used. The mobile phase consisted of a gradient between A: CH_3_OH-CH_3_CN (9:1, v/v) 75% - H_2_O 25%; B: CH_3_OH-CH_3_CN (9:1, v/v) and C: ipOH, all containing ammonium acetate (5mM). An ESI probe operated in negative mode was used for sample ionization. The mass spectrometer parameters were as follows: capillary voltage: 2.9kV; cone voltage: 70V; desolvation temperature: 400°C; desolvation gas flow: 1000L/Hour; cone gas flow: 150L/Hr; nebuliser: 6bar.

For the cardiolipins, a Nucleosil C8 column 150x4mm, 5 µm (Macherey-Nagel) was used. The mobile phase was a gradient between A: CH_3_OH-CH_3_CN (9:1, v/v) 75% - H_2_O 25%; B: CH_3_OH-CH_3_CN (9:1, v/v) and C: IpOH, all containing ammonium acetate (5 mM). An ESI probe operated in negative mode was used for cardiolipin ionization.

The obtained data were adjusted to the relative lipid content determined *via* labeling the lipids with FM 4-64, as mentioned above. The relative quantification of the lipids was based on the ratio between the area under the curve (AUC) of the lipid species and the AUC of the respective internal standard.

**Determination of the MVs fatty acid composition**

The total lipids were extracted from the purified MVs isolated from planktonic and biofilm P. aeruginosa using chloroform/methanol/water (2:2:1.8; v:v:v) (6), and the lipid extracts were dried under nitrogen. For fatty acid analysis, the extracted fatty acids were converted to fatty acid methyl esters according to the established protocol (7). Briefly, the extracted lipids were incubated at 70 C° with 0.5 ml of 0.1 mol/l KOH in methanol for 1 hour, followed by a 15 minutes incubation in 0.2 ml of 1.2 mol/l HCl in methanol. Then, the fatty acid methyl esters (FAME) were extracted by 1 ml hexane, and they were separated via gas-liquid chromatography.

The chromatograph (GC Trace-1310, Thermo Scientific, Italy) was equipped with an RT2560 capillary column (100 m × 0.25 mm internal diameter, 0.2 μm film thickness; Restek) and a flame ionization detector (FID, Thermo Quest). H2 was used as carrier gas at constant pressure (200 kPa). The FID was continuously flowed by H2 (35 ml/min) and air (350 ml/min) and kept at a constant temperature of 255°C. The temperature program was as follows: an initial temperature of 80°C, which increased at 25°C/min up to 175°C, a holding temperature of 175°C during 25 min, a new increase at 10°C/min up to 205°C, a holding temperature of 205°C during 4 min, a new increase at 10°C/min up to 215°C, a holding temperature of 215°C during 25 min, the last increase at 10°C/min up to 235°C and a final holding temperature of 235°C during 10 min (8).

**Compared with planktonic bacteria, biofilm *P. aeruginosa* has a distinct membrane phospholipid composition and differential expression of fatty acid biosynthesis genes**

The lipids from the isolated inner membranes were subjected to mass-spectrometry analysis (Figure S1). As reported in the literature, PE is the most abundant phospholipid in the *P. aeruginosa* membrane (9, 10). In regard to the relative abundance of fatty acids, the membranes of the planktonic bacteria have higher proportions of PE with unsaturated fatty acid chains than the membranes of the biofilm *P. aeruginosa* (Figure S1-A). Conversely, PE species having saturated fatty acid chains are more abundant in the membranes of biofilm *P. aeruginosa* compared with those of planktonic bacteria (Figure S1- B). Moreover, the relative abundance of PE species was not significantly different in the membrane of planktonic *P. aeruginosa* compared with that of the biofilm bacteria (Figure S1- C). Additionally, lipid analysis showed, regardless of their fatty acyl chains, PG species are present in a significantly higher proportion in the membrane of biofilm *P. aeruginosa* than in the membrane of the planktonic bacteria (Figure S1-A, B, and C). The enrichment of the membrane of biofilm *P. aeruginosa* with saturated fatty acids and PG agrees with the previous studies reporting that decreased membrane fluidity is observed in the biofilm mode of growth (11, 12).

Besides, the genes encoding the long-chain fatty acid synthesis enzymes, i.e., Fad D1 and Fad D2, which contribute to the changing the chain length of fatty acids via utilization of exogenous fatty acids, are significantly overexpressed in the biofilm bacteria. In contrast, the genes encoding desaturases enzymes (Des A and Des B) are notably downregulated in the biofilm bacteria relative to that in planktonic cells (Figure S1- D).

**Comparative study of the phospholipid composition of the MVs**

Despite the membranes of planktonic *P. aeruginosa* being significantly enriched with phospholipids containing unsaturated fatty acids relative to the membranes of the biofilm bacteria (Figure S1-A), unsaturated fatty acids were not present in a considerably higher proportion in the MVs of the planktonic bacteria in comparison with that in the biofilm bacteria (Figure S3- A). However, phospholipids with saturated fatty acids occupied greater proportions in MVs of the biofilm bacteria than in MVs of planktonic bacteria (Figure S3- B). Further, comparison of the polar heads of phospholipids showed that PE is present in roughly equal relative amounts in the MVs of planktonic and biofilm bacteria (Figure S3- C). PG is present at a higher level in the MVs of biofilm *P. aeruginosa* than in those of planktonic bacteria (Figure S3- C). The difference in the relative proportion of CL in MVs of the planktonic bacteria vs. MVs of biofilm bacteria is not significant (Figure S3- C).

**Lifetime map of BODIPY-C10 over the *P. aeruginosa* membrane and the spheroplasted bacteria**

To ensure that the BODIPY-C10 incorporates to the inner membrane of *P. aeruginosa*, we prepared the spheroplasts of the bacteria and labeled them with the probe. Bacterial cultures were grown in LB for 12 hours. 1ml of culture was centrifuged (2978 g for 20 minutes at 4°C (Eppendorf 5810 R centrifuge; A-4-62 rotor)). The pellet (~10^9^ CFU/mL) was resuspended in a buffer containing 1 M sucrose, 10 mM Tris-HCl, and 150 μg/mL lysozyme. The suspension was kept on ice for 5 minutes, and then EDTA solution (1.5 M, pH: 7.5) was slowly added to the mixture. After 3 minutes, the formation of the spheroplast was confirmed under an optical microscope. We had the bacteria and the spheroplasts in the cell suspension simultaneously. Then, the suspension were pelleted via centrifugation (2978 g for 20 minutes at 4°C (Eppendorf 5810 R centrifuge; A-4-62 rotor)). The labeling of the spheroplasts and slide preparation steps performed according to the protocol used for the bacteria.

We observed that the spheroplasts are labeled with the probe (Figure S4-A). Additionally, and the probe has roughly equal lifetime, when it incorporates to the inner membrane of the bacterial cell in comparison with the spheroplasted bacteria (Figure S4-B). A previous study on the localization of the BODIPY-C10 in the membrane of Gram-negative bacteria showed; the probe’s lifetime showed similar values when the probe labeled *Escherichia coli* cells compared to that labeling spheroplast of *E. coli*. This observation suggests the probe localizes in the lipids of the inner membrane of bacteria (4).

**Effect of MVs lipid extracts on membrane viscosity of the planktonic *P. aeruginosa***

Total lipids were extracted from 10^8^ particles of the MVs isolated from planktonic and biofilm *P. aeruginosa* using chloroform/methanol/water (2:2:1.8; v:v:v) according to Bligh and Dyer lipid extraction protocol (6). Then, *P. aeruginosa* was inoculated in the media (10^5^ CFU/mL) supplemented with the extracted lipids dissolved in methanol. After 4 hours of growth, the cells were washed with PBS. The bacterial cells were prepared for FLIM and the viscosity analysis according to the abovementioned protocol. Interestingly, this experiment showed roughly the same trend of effect compared to the investigation related to the effect of MVs of the biofilm bacteria in altering the membrane viscosity of the planktonic bacteria (Figure *4-in the manuscript)*. Figure S5 shows that lipids extracted from the MVs of planktonic and biofilm *P. aeruginosa* can significantly increase the viscosity of the bacterial membrane compared to that in non-treated control. However, the effect of lipids of MVs produced by the biofilm bacteria in changing the bacterial membrane's viscosity is more pronounced than that of the lipids extracted from the MVs of the planktonic bacteria.

**FLIM observations and analysis**

For FLIM analysis, first, histograms of the distribution of each lifetime over the pixels were plotted (Figure S6). The fluorescence lifetime of BODIPY-C10 is directly correlated to the viscosity of the environment in which it is embedded (2). The precise analysis of the FLIM fitting showed that the fluorescence decay of the pixels of each image were fit to a bi-exponential decay model (goodness of fit; chi^2^: 1.12 to 1.85), indicating the presence of two-lifetime components in the images (Example of decay fitted curves in Figure S7). Previous study of the localization of BODIPY-C10 in the membrane of Gram-negative bacteria showed that this probe mostly localizes in the lipids of the inner membrane (4). Given the inner membranes of bacteria are composed of phospholipids, the probe incorporates differentially into phosphate polar heads of phospholipids and their fatty acyl chains. The hydrophilic polar heads are considered to be more fluid than the hydrophobic tail; hence, the observation of two-lifetime components is related to the rotation of the probe (molecular rotor) in the two environments with different fluidity. However, the longer lifetime component best represents the membrane fluidity (4, 13). Similarly, in this study, two lifetime components were observed, one with a short lifetime (0.36 to 0.58 ns) and another with a longer lifetime (1.5 to 3.5 ns). We took the average of the long-lifetime component for illustration of the fluidity map (Figure S8) and calculation of viscosity.

The fluorescence lifetime of a molecular rotor (τ_f_) is correlated to the viscosity of its microenvironment (14) and is determined by the rates of radiative and non-radiative decay of the fluorophore via (Equation 1);

Equation 1: τ_f_ = $\frac{1}{Radiative decay rate+Non-Radiative decay rate}$

The correlation between lifetime and viscosity (η) was determined by Foster-Hoffman. Accordingly, the viscosity is calculated via the Foster-Hoffman equation (Equation 2), where z and α are constants (15). Hence, the placement of the probe in a highly viscous microenvironment leads to a longer fluorescence lifetime and vice versa.

Equation 2: zηα= $\frac{Radiative decay rate}{Radia tive decay rate+Non-Radiative decay rate}$

**FLIM imaging and analysis**

*FLIM imaging:* Bacteria in Ibidi chamber slide (IBIDI™ GmbH, Germany) were observed on the LSM980 multiphoton microscope (Zeiss, Germany) equipped with a time-correlated single-photon counting (TCSPC) FLIM module (PicoQuant, Germany) for high-resolution microscopy. BODIPY-C10 was excited with Coherent (Chameleon Discovery) pulsed laser (80 MHz) at 800 nm. The emission was recorded using a bandpass filter of 505-545 nm at a resolution of 512×512 pixels. Recording of the FLIM images took 3 to 5 minutes, depending on the photon collection. A minimum of 1000 photons in the brightest pixel was required before stopping the FLIM acquisition.

*FLIM analysis:* The analysis of the FLIM images was performed with SymPhoTime64 software (PicoQuant, Germany). The lifetime data were converted into viscosity data using the viscosity-lifetime calibration equation, which was established by measuring the fluorescence lifetime of BODIPY C10 in several methanol/glycerol mixtures with known viscosities according to the following equation (η: viscosity (cP) and τ: lifetime (ns)) (4):

Log η =$\frac{Log \tau+0.75614}{0.4569}$

Data illustrated in the box plots show that the fluorescence lifetime of the probe in the membrane of the sessile bacteria is significantly longer than in that of planktonic bacteria. Hence, sessile bacteria have a less fluid membrane than planktonic bacteria (Figure S8). To ensure that the interaction of BODIPY-C10 and poly-L-Lysin do not bias the recorded lifetime, FLIM imaging was also performed on chambers covered with 0.1% poly-L-Lysine alone (without bacteria) and only incubated with the probe, strictly following the protocols for bacterial preparations.

**References**

1. Gao J, Zhao B, Wang M, Serrano MA, Zhuang J, Ray M, Rotello VM, Vachet RW, Thayumanavan S. 2018. Supramolecular assemblies for transporting proteins across an immiscible solvent interface. J Am Chem Soc 140:2421-2425.

2. Kuimova MK, Yahioglu G, Levitt JA, Suhling K. 2008. Molecular rotor measures viscosity of live cells via fluorescence lifetime imaging. J Am Chem Soc 130:6672-6673.

3. Woodcock EM, Girvan P, Eckert J, Lopez-Duarte I, Kubánková M, van Loon JJ, Brooks NJ, Kuimova MK. 2019. Measuring intracellular viscosity in conditions of hypergravity. Biophys J 116:1984-1993.

4. Mika JT, Thompson AJ, Dent MR, Brooks NJ, Michiels J, Hofkens J, Kuimova MK. 2016. Measuring the viscosity of the Escherichia coli plasma membrane using molecular rotors. Biophys J 111:1528-1540.

5. Contreras I, Shapiro L, Henry S. 1978. Membrane phospholipid composition of Caulobacter crescentus. J Bacteriol 135:1130-1136.

6. Bligh EG, Dyer WJ. 1959. A rapid method of total lipid extraction and purification. Can J Biochem 37:911-917.

7. Schneider A-C, Beguin P, Bourez S, Perfield JW, Mignolet E, Debier C, Schneider Y-J, Larondelle Y. 2012. Conversion of t11t13 CLA into c9t11 CLA in Caco-2 cells and inhibition by sterculic oil. PLoS One 7:e32824.

8. Ferain A, Bonnineau C, Neefs I, Rees JF, Larondelle Y, De Schamphelaere KA, Debier C. 2016. The fatty acid profile of rainbow trout liver cells modulates their tolerance to methylmercury and cadmium. Aquat Toxicol 177:171-181.

9. Zhang Y-M, Rock CO. 2008. Membrane lipid homeostasis in bacteria. Nat Rev Microbiol 6:222-233.

10. Tashiro Y, Inagaki A, Shimizu M, Ichikawa S, Takaya N, Nakajima-Kambe T, Uchiyama H, Nomura N. 2011. Characterization of phospholipids in membrane vesicles derived from Pseudomonas aeruginosa. Biosci Biotechnol Biochem 75:605-607.

11. Benamara H, Rihouey C, Jouenne T, Alexandre S. 2011. Impact of the biofilm mode of growth on the inner membrane phospholipid composition and lipid domains in Pseudomonas aeruginosa. Biochim Biophys Acta Biomembr 1808:98-105.

12. Dubois-Brissonnet F, Trotier E, Briandet R. 2016. The biofilm lifestyle involves an increase in bacterial membrane saturated fatty acids. Front Microbiol 7:1673.

13. Kashirina AS, López-Duarte I, Kubánková M, Gulin AA, Dudenkova VV, Rodimova SA, Torgomyan HG, Zagaynova EV, Meleshina AV, Kuimova MK. 2020. Monitoring membrane viscosity in differentiating stem cells using BODIPY-based molecular rotors and FLIM. Sci Rep 10:1-12.

14. Levitt JA, Chung PH, Kuimova MK, Yahioglu G, Wang Y, Qu J, Suhling K. 2011. Fluorescence anisotropy of molecular rotors. Chempluschem 12:662-672.

15. Dent MR, López-Duarte I, Dickson CJ, Geoghegan ND, Cooper JM, Gould IR, Krams R, Bull JA, Brooks NJ, Kuimova MK. 2015. Imaging phase separation in model lipid membranes through the use of BODIPY based molecular rotors. Phys Chem Chem Phys 17:18393-18402.
