# Supplementary material for: Contribution of Membrane Vesicle to Reprogramming of Bacterial Membrane Fluidity in Pseudomonas aeruginosa
Source: mSphere. 2022 May 23;7(3):e00187-22. doi: 10.1128/msphere.00187-22 (PMC9241526; doi:10.1128/msphere.00187-22)
Supplement: TABLE S1 [file msphere.00187-22-s0002.docx]

**Primers**

Table S1 Primer sequences

| Gene | Forward primer (5' to 3') | Reverse Primer (5' to 3') |
| --- | --- | --- |
| *Des A* | GATGGTATGGATTCCGTTCTG | GGTGTGGTGGTTGTTGTG |
| *Des B* | AACCATATCCACCACACCTAC | TAGCAGCGGCATGAGTTC |
| *Fad D1* | AATGTGTATCCGAACGAACTG | TTGACCACCACGAAGACC |
| *Fad D2* | CGCCACCAGTTCAAGGAC | ACTTCACCACGCTGTTCAC |
